# Load-Deflection Behavior of Over- and Under-Reinforced Concrete Beams with Hybrid FRP-Steel Reinforcements

**DOI:** 10.3390/ma14185341

**Published:** 2021-09-16

**Authors:** Saruhan Kartal, Ilker Kalkan, Ahmet Beycioglu, Magdalena Dobiszewska

**Affiliations:** 1 Department of Civil Engineering, Faculty of Engineering and Architecture, Kırıkkkale University, Kırıkkale 71450, Turkey; saruhankartal@kku.edu.tr; 2 Department of Civil Engineering, Adana Alparslan Türkeş Science and Technology University, Adana 01250, Turkey; abeycioglu@atu.edu.tr; 3 Faculty of Civil and Environmental and Architecture, Bydgoszcz University of Science and Technology, Al. Prof. S. Kaliskiego 7, 85-796 Bydgoszcz, Poland; magdalena.dobiszewska@utp.edu.pl

**Keywords:** effective moment of inertia, tension stiffening, flexural cracking, FRP rupture, fully cracked section, first-cracking moment

## Abstract

The present study pertains to the load-deflection behavior and cracking moments of concrete beams with hybrid FRP-steel reinforcement. Under and over-reinforced hybrid beams were tested for failure along with reference beams with only steel or FRP reinforcement. The first-cracking moments of the beams were estimated analytically by using different uncracked moments of the inertia and modulus of rupture definitions. The uncracked moment of inertia definitions include the gross and uncracked transformed moments. The adopted modulus definitions are comprised of the experimental values from tests on prisms and the analytical values from the equations in different concrete codes. Furthermore, analytical methods were developed for estimating the deflections of concrete beams with hybrid FRP-steel or only FRP reinforcement. Two different types of elastic moduli, namely the secant modulus corresponding to the extreme compression fiber strain and the ACI 318M-19 modulus, were used in deflection calculations. Closer estimates were obtained by using the secant modulus, particularly in hybrid-reinforced beams. In the post-yielding region of the steel tension reinforcement, the deflection estimates were established to lay in closer proximity to the experimental curve when obtained by adding up the deflection increments instead of directly calculating the total deflections from the elastic curve equation. Accurate estimation of the cracking moment was found to be vital for the close prediction of deflections.

## 1. Introduction

The loss of the reinforcing bar (rebar) cross-section and the reduction in the bond strength of rebars in concrete due to the formation of debonding cracks are among the most deleterious effects of reinforcement corrosion on reinforced concrete (RC) members and structures. Reinforcement corrosion is a more pronounced problem in structures subjected to aggressive environmental conditions, including but not limited to the presence of chlorides, sulphates, and other active chemicals. Furthermore, RC members are always liable to cracking due to chemical and physical events, such as alkali–silica reactions and the carbonation and restrained shrinkage of concrete. These cracks form paths for the aggressive agents and air to reach the steel rebars, which can exacerbate the corrosion problem during the service lives of structures. The reinforcement corrosion leads to a reduction in the load capacities of members as well as serviceability problems, i.e., increased deformations and crack widths. Extensive research has been conducted on protection methods for reinforcement corrosion, including surface coating of rebars (epoxy coating, galvanizing, zinc coating, and copper cladding), cathodic protection, and the use of organic and inorganic admixtures in concrete. Recently, a new design concept started being implemented, which is the use of non-corroding polymeric (FRP) materials as reinforcement rather than adopting precautions against steel corrosion. Although there has been a proliferation of studies on the use of composite materials in strengthening concrete beams [1,2,3], the direct use of polymers as a concrete reinforcement is a fairly new concept. Despite their major advantages, various drawbacks arise when concrete members are reinforced with only FRP bars. Ductility problems associated with the brittle material properties and serviceability problems originating from the low elastic modulus values of FRP materials are among the leading shortcomings of the use of FRP as the sole reinforcement material in concrete flexural members (beams and slabs). Furthermore, FRP bars cannot be utilized in concrete columns since they are liable to buckling even under low compressive forces as a result of their low elastic modulus values. The ductility and serviceability concerns receive particular attention in the design of FRP RC members [4].

The simultaneous presence of FRP and steel bars in the tension zone developed as an efficient reinforcement scheme for concrete flexural members. The so-called hybrid FRP-steel RC members offer an effective solution not only to ductility and serviceability problems of FRP RC members but also to the reductions in the strength and stiffness of steel RC beams due to steel corrosion. By locating the FRP bars closer to the corners and the steel bars at the inner portion of the section, the liability of the steel reinforcement to corrosion can be reduced in the hybrid design. The yielding of steel bars in the tension zone provides the flexural members with greater ductilities compared to the FRP RC ones no matter if the FRP bars rupture before the concrete crushing (under-reinforced in terms of FRP reinforcement) or concrete crushes before the FRP rupture (over-reinforced in terms of FRP reinforcement). Furthermore, the higher elastic modulus of steel reduces the member deformations and crack widths at service load levels.

A great majority of the previous studies in the literature on hybrid FRP-steel RC beams [5,6,7,8,9,10,11,12,13,14] focused on the bending moment capacities, failure modes, and ductilities of these beams. The load capacities and ductility values of beams, obtained from the load-deflection curves, were compared to each other to investigate the variation of these measures with varying proportions of FRP and steel within the tension reinforcement. The previous researchers also developed analytical methods for estimating load capacities of hybrid RC beams. Nevertheless, none of the previous studies known to the authors aimed at developing analytical methods for estimating the load-deflection curves and cracking moments of these beams. Furthermore, the previous studies mostly focused on over-reinforced hybrid RC beams. In other words, the flexural capacity was targeted to be reached as a result of concrete crushing rather than of FRP rupture. Compression failure, i.e., over-reinforced beam design, is suggested by various international FRP RC codes [4,15] considering the FRP rupture takes place more suddenly and in a more brittle manner compared to the concrete crushing. However, designers are not obliged to design RC beams as over-reinforced in the presence of steel bars in the tension zone, in addition to FRP bars. Steel yielding always precedes FRP rupture and concrete crushing in hybrid RC beams and a moderate ductility level is always attained whether the beam is under-reinforced or over-reinforced in terms of the FRP reinforcement. Consequently, the lack of experimental data on under-reinforced hybrid RC beams in the literature is a vital handicap for the researchers seeking to develop a comprehensive analytical method for estimating the load-deflection behavior, ductilities, and cracking moments of hybrid steel-FRP RC beams.

The present study pertains to developing analytical methods for the load-deflection behavior and initial cracking moments of hybrid FRP-steel RC beams. In order to conduct a comprehensive analysis, under and over-reinforced hybrid RC beams were tested together with reference beams with only steel or only FRP reinforcement. The authors tested both over and under-reinforced hybrid RC beams for two main reasons:

- Accurate deflection and cracking moment calculations strongly depend on the accurate determination of the material properties of concrete and reinforcement. In none of the studies in the literature were all needed mechanical properties of concrete and reinforcement encountered. The authors determined the compressive strength of concrete from cylinder tests and the modulus of rupture from the prism tests. Furthermore, the yielding stress of steel, the rupture strength, and elastic modulus of FRP were determined from axial tension tests. The FRP bar samples were tested according to a special procedure to reach reliable tensile strength and elastic modulus values. With the help of all these material tests, the authors could reach more reliable conclusions on the proposed and available analytical methods. Due to the lack of all these measured material properties, the authors could not rely on test results of the existing over-reinforced beams in the literature.
- Unlike the previous studies, the authors are of the opinion that RC beams with hybrid FRP-steel reinforcement can be designed under-reinforced in terms of FRP reinforcement. The required ductility of the beam is achieved with the help of the yielding of steel reinforcement, even if the FRP bar ruptures before the concrete crushing. The previous researchers preferred concrete crushing to precede FRP rupture, as concrete crushing is more ductile than FRP rupture. However, they ignored the fact that the required ductile behavior of a hybrid FRP-steel RC beam is already reached with the yielding of steel before the concrete crushing or FRP rupture. Hence, the authors also tested under-reinforced beams in this study.

Within the scope of the present study, first, the effective moment of inertia expressions in the literature are discussed. Next, the experimental stage of the study is summarized together with the failure modes and load-deflection behavior of the specimens. Next, analytical methods for estimating the first-cracking moments of beams with different types of reinforcement are introduced and the estimates from these methods are compared to the experimental cracking moments. The modulus of rupture values from flexural tests on prismatic concrete specimens were used in the cracking moment calculations as well as the analytical values from the formulations in the international concrete codes. The cracking moment calculations were realized for two types of uncracked cross-sections, namely uncracked transformed and gross. Finally, new incremental approaches were proposed for hybrid FRP-steel and FRP RC beams to increase the precision of the deflection estimates. The accuracy of the estimates was evaluated for steel RC, FRP RC, over-reinforced hybrid RC, and under-reinforced hybrid RC beam groups, separately. Different modulus of elasticity and uncracked moment of inertia definitions were considered in the analytical deflection calculations.

## 2. Existing Effective Moment of Inertia Expressions

The low modulus of elasticity of FRP manifests the need for making accurate deflection predictions in FRP and hybrid FRP-steel RC beams so that these deflections do not become critical under service loads. Most of the studies on deflections of FRP RC beams focused on examining the existing or developing new effective moment of inertia expressions. Most of these effective moment of inertia expressions originate from the well-known Branson [16] equation:

(1) $$I_{e}={\left(\frac{M_{cr}}{M_{a}}\right)}^{m}\;I_{g}+\left[1-{\left(\frac{M_{cr}}{M_{a}}\right)}^{m}\right]\;I_{cr}\leq \;I_{g}$$

where *M_cr_* and *M_a_* are the cracking and applied moments of the beam, respectively, while *I_g_* and *I_cr_* are the gross and fully cracked moments of inertia, respectively. Branson [16] proposed a value of three for the power m in the equation.

The accuracy of predictions from Equation (1) is known to depend on the uncracked-to-cracked moment of the inertia (*I_g_*/*I_cr_*) ratio of a beam. This ratio, in turn, depends on the tension reinforcement ratio (ρ) and reinforcement-to-concrete modular ratio (*n*). Bischoff [17] pointed out that Equation (1) closely estimates the effective moment of inertia values of RC beams when the *I_g_*/*I_cr_* ratio is smaller than three. Conversely, in concrete beams with FRP reinforcement, the *I_g_*/*I_cr_* ratio has a value between 5 and 25, and therefore Equation (1) with m = 3 overestimates the tension-stiffening effect, considering the deflections of FRP RC beams are underestimated if Equation (1) is adopted in the deflection calculations. The low modulus of elasticity values of FRP materials and the use of smaller tension reinforcement areas in FRP RC beams due to the high tensile strength values of FRP bars were held responsible for the *I_g_*/*I_cr_* ratio exceeding three and the Branson [16] formula not being applicable to FRP RC beams.

Several researchers proposed different equations that account for the reduced tension-stiffening contribution in FRP RC beams. The effective moment of inertia equation in ACI 440.1R-96 [18] was originally developed by Faza and GangaRao [19]. This equation, as given below, is based on the assumption that an FRP RC beam is in the fully cracked condition between the loading points and in the partially cracked condition in shear spans. *I_m_* in this equation is denoted as the modified effective moment of inertia, which is a geometric weighted average of *I_cr_* and *I_e_*:


(2) $$I_{m}=\frac{23I_{cr}I_{e}}{8I_{cr}+15I_{e}}$$

*I_e_* in this equation is calculated from Equation (1). Toutanji and Saafi [20] modified the original Branson’s [16] equation in compliance with the modulus of elasticity of FRP and reinforcement ratio, and proposed the following equations:


(3) Ie=McrMa6−10ρfEfEs Ig+1−McrMa6−10ρfEfEs Icr if EfEsρf<0.3McrMa3 Ig+1−McrMa3 Icr if EfEsρf≥0.3≤Ig

*E_f_* and *E_s_* in these equations are the modulus of elasticity values of FRP and steel, respectively, and *ρ_f_* is the reinforcement ratio. Alsayed et al. [21] proposed two simple and easily applicable methods for the estimation of service-load deflections of FRP RC beams. Based on regression analyses on RC beams with GFRP reinforcement, a power of *m* = 5.5 was proposed for Branson’s [16] formula. Another set of equations (Equation (4)) was also developed by setting a relationship between the *I_e_*/*I_cr_* ratio and *M_a_*/*M_cr_* ratio. The service-load deflections of FRP RC beams were compared to the analytical estimates from Equation (4) and other effective moment of inertia equations in the literature. This comparison indicated that Equation (4) gives much closer estimates to the actual values as compared to the other existing formulations.


(4) $$I_{e}=\left\{\begin{array}{cc} \left(1.40-\frac{2}{15}\cdot \frac{M_{a}}{M_{cr}}\right)I_{cr} & \mathrm{if}\;1.0<\frac{M_{a}}{M_{cr}}<3.0\\ I_{cr} & \mathrm{if}\;\;\;\frac{M_{a}}{M_{cr}}>3.0 \end{array}\right\}$$

Benmokrane et al. [22] introduced reduction terms to Equation (1), as follows:


(5) $$I_{e}={\left(\frac{M_{cr}}{M_{a}}\right)}^{3\;}\frac{I_{g}}{\beta }+\alpha \left[1-{\left(\frac{M_{cr}}{M_{a}}\right)}^{3}\right]\;I_{cr}\leq \;I_{g}$$

The *α* and *β* terms in this expression are taken as 0.84 and 7, respectively. The deflection estimates obtained by using Equation (5) were shown to be in much closer agreement with the experimental results of FRP RC beams as compared to other analytical formulations. Gao et al. [23] modified Equation (5) to reach higher degrees of agreement with the experimental results:


(6) $$I_{e}={\left(\frac{M_{cr}}{M_{a}}\right)}^{3\;}\beta_{d}I_{g}+\left[1-{\left(\frac{M_{cr}}{M_{a}}\right)}^{3}\right]\;I_{cr}\leq \;I_{g}$$

The term *β_d_* in this equation reflects the effects of the modulus of elasticity and the bonding properties of an FRP bar on beam deflections and it is calculated from the following equation: 


(7) $$\beta_{d}=\alpha_{b}\cdot \left(\frac{E_{f}}{E_{s}}+1\right)$$

A value of 0.5 was assigned by Gao et al. [23] to the term *α_b_* for GFRP bars. Many international FRP RC codes [24,25] recommend the use of this equation for predicting the deflections of beams. Upon finding out that Equation (7) still underestimates the deflections of certain FRP RC beams, Yost et al. [26] modified the *α_b_* term in the equation by expressing it in terms of the reinforcement ratio as follows:

(8) $$\alpha_{b}=0.064\cdot \left(\rho_{f}/\rho_{fb}\right)+0.13$$

where *ρ_fb_* is the balanced reinforcement ratio of the beam. Different versions of the ACI 440.1R code [4,27] also recommend the use of Equation (6) but present a different equation for *β_d_* in terms of the reinforcement ratio: 


(9) $$\beta_{d}=0.2\cdot \left(\rho_{f}/\rho_{fb}\right)$$

Finally, Bischoff [17] developed a new equation that can be used in all RC beams irrespective of the modulus of the elasticity of reinforcement:


(10) $$\frac{1}{I_{e}}={\left(\frac{M_{cr}}{M_{a}}\right)}^{2}\frac{1}{I_{g}}+\left[1-{\left(\frac{M_{cr}}{M_{a}}\right)}^{2}\right]\frac{1}{I_{cr}}$$

The most important difference between Equations (1) and (10) is that the uncracked portions between the discrete cracks and the cracked portions are modeled with springs in series. The original Branson [16] equation relies on the assumption that the uncracked and cracked portions should be modeled with springs in parallel. Nevertheless, this model does not correctly reflect the stress and loading conditions of adjacent uncracked and cracked portions of a beam. These portions do not share the internal force in the tension zone as in the springs in parallel but they do resist the same tensile force as in the springs in series. The inaccurate modeling with springs in parallel clarifies the reason for the Branson [16] equation to overestimate the tension-stiffening effect. 

The Bischoff [17] equation can be applied to all steel RC and FRP RC beams irrespective of the *I_g_*/*I_cr_* ratio [28]. The studies in the literature depicted that this equation can closely predict the experimental deflections of hybrid FRP-steel RC beams [7,10,12,13]. For all these reasons, the Bischoff [17] effective moment of inertia equation was used in the present study.

## 3. Experimental Study

### 3.1. Test Specimens and Material Properties

A total of 25 beams with only steel, BFRP, GFRP, hybrid BFRP-steel, and hybrid GFRP-steel reinforcement were tested for failure under four-point bending. The beams were classified into three groups based on their load capacities and failure modes (Table 1). The steel RC and FRP RC beams in each group were adopted as the reference beams. The steel RC reference beam in each test group was under-reinforced and the FRP RC reference beam was over-reinforced. 

Testing beams with approximately identical bending capacities enabled the researchers to investigate the influence of variation in the proportions of steel and FRP within the tension reinforcement on the beam ductility, energy absorption capacity, and load-deflection behavior.

**Figure 1:**
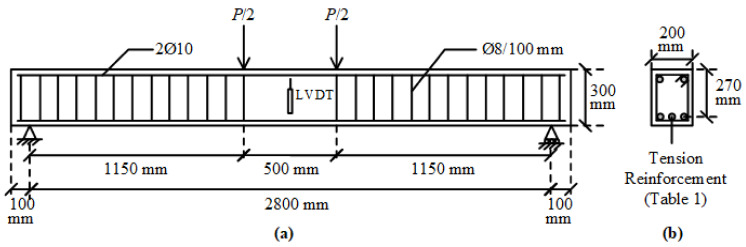
Reinforcement details and dimensions of the test beams. (**a**) Elevation and (**b**) cross-section.

The beam notations reflect the number and types of tension bars in each beam. The capital letters “B”, “G”, and “S” correspond to the BFRP, GFRP, and steel rebars, respectively. The number after each capital letter shows the number of that type of bar. For instance, specimen B2S3 is the beam with two BFRPs and three steel bars in the tension zone, while beam S5 denotes the reference beam with five steel tension bars. The details and layout of the reinforcement in each specimen are illustrated in Figure 1 and Table 1.

The first group of beams was designed as over-reinforced in terms of FRP reinforcement. In other words, concrete crushing preceded the rupture of FRP bars, yet occurred after the yielding of steel bars. All of the beams in this group contained a total of five tension bars. Two types of FRP bars, namely BFRP and GFRP, were used in the first set alongside steel bars. The second set of beams, in contrast, were reinforced with six tension bars of two types (GFRP and steel). This group was designed again as over-reinforced. The third group had only a total of three tension bars of three types (BFRP, GFRP, and steel) to provide an under-reinforced beam design, i.e., a tension-controlled mode of failure. In this type of failure, FRP rupture takes place before the concrete crushing, yet occurred after the yielding of steel bars. 

Specimen B5 in the last group contained five BFRP bars, unlike the remaining beams in the group, considering the manufacturer could not provide a sufficient number of bars with a 8.68-mm diameter. For this reason, a beam with five Ø5.3 bars and with the same tensile reinforcement area as the three Ø8.68 bars was constructed as the reference beam.

**Figure 2:**
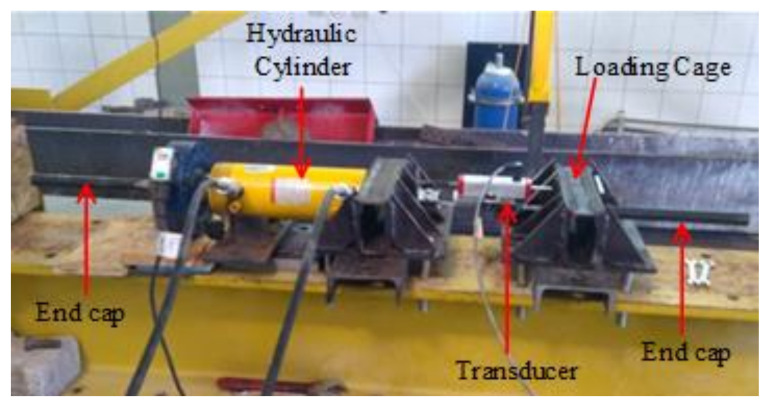
Setup for axial tension tests on FRP bar samples.

Table 1 tabulates the mechanical properties of FRP bars from the material tests. These measured values were determined from axial tension tests on bar samples with grout-filled tubular steel end caps, as suggested by ASTM D7205/D7205M-06 [29]. A special loading mechanism, composed of a hydraulic cylinder and a loading cage, was formed to test the bar samples. The load applied by the cylinder was transmitted to the tubular end caps with the help of the loading cage (Figure 2). Although all of the GFRP bars in the specimens were purchased from the same supplier, the bars in the first group of beams (GFRPType-1) had different mechanical properties than the ones in the other two groups (GFRPType-2). These two types of GFRP bars were not produced simultaneously, i.e., from the same batch. The mechanical properties of FRP bars are known to have significant variations due to various factors, including slight deviations of the fibers from the axial direction as well as undesired but unavoidable variations in the contents of the polymer matrix and fibers. Three BFRP, four GFRPType-1, and four GFRPType-2 bar samples were tested and both the mean values and coefficients of variation of the tests on each bar type are given in Table 1.

The longitudinal and transverse steel reinforcement in the beams were of grade 420 with an average measured yielding stress value of 470 MPa according to the material tests. Each test beam was provided with an adequate shear strength by using two-legged Ø8/100 mm stirrups, with the exception of the central constant-moment, i.e., the zero-shear zone of the beam. In the second and last groups of beams, the compression reinforcement was not extended to the central zone. Unlike these two groups, the workers forgot to cut the compression bars at the interface of the shear span and at the central constant-moment portion in the first set. The researchers noticed this mistake after the concrete cast. As expected, the presence of compression bars in this region, with no lateral support from the stirrups, resulted in the buckling of these bars and hence the premature crushing of the compression concrete. 

The effect of compression bar buckling on the concrete crushing was identified by conducting two additional tests on a beam (G1S4) with and without compression bars in the central region. The concrete strain at the crushing was measured in these tests with the help of strain gauges. Accordingly, the crushing of concrete initiates at strain values of 0.0037 and 0.0030 in the absence and presence of unbraced compression reinforcement in the central portion. These strain values were used in the analytical calculations of the three groups of beams. 

The second and third groups of beams were cast in a single pour and the first group in a separate pour than the other two groups. The concrete compressive strength of each pour was determined from material tests on 150 mm × 300 mm and 100 mm × 200 mm cylinder tests and the size effect was taken into account when calculating the mean measured compressive strength of concrete as 31.28 and 30.49 MPa for the first and remaining (second and third) groups of beams, respectively. For an accurate estimation of the cracking moments and deflections of beams, the modulus of rupture, i.e., the flexural tensile strength, values of the concrete mixes were also measured with the help of four-point bending tests on 150 × 150 × 600-mm prismatic samples [30]. Accordingly, the modulus of rupture values of concrete batches were obtained as 3.55 and 3.25 MPa for the first and remaining groups of beams, respectively.

### 3.2. Experimental Setup

**Figure 3:**
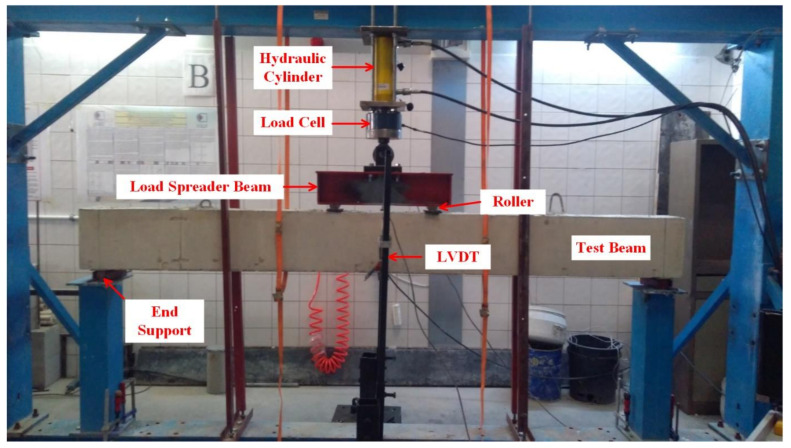
Test setup.

The test beams were simply supported at the ends and subjected to two-point loading (Figure 1 and Figure 3). Each loading point was at a distance of 1150 mm from the end support, leaving a distance of 500 mm between the loading points. The clear distance between the end supports was 2800 mm (Figure 1). The vertical deflections at mid-span were measured with the help of two LVDTs, connected to the front and rear faces of the beam. In this way, the twisting rotations in the beam during loading were attempted to be detected in the case of any undesired eccentricities in the applied load. The mid-span deflections were calculated from the average of the measured values from the two LVDTs. The load measurements from the load cell and the deflection measurements from the LVDTs were acquired by a data acquisition system and saved to a computer.

## 4. Experimental Results and Discussions

**Figure 4:**
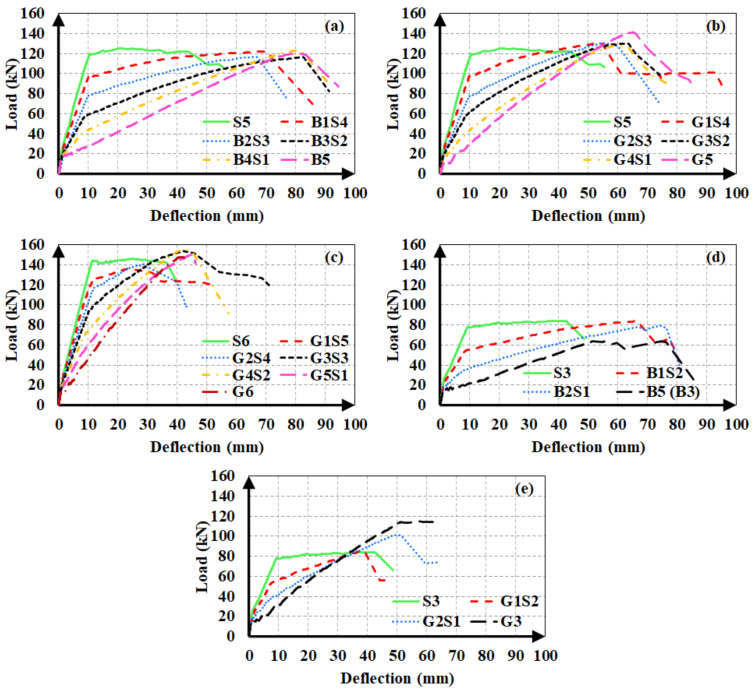
Load-deflection curves of the (**a**) first group with BFRP; (**b**) first group with GFRP; (**c**) second group; (**d**) third group with BFRP; and (**e**) third group with GFRP.

The experimental load-deflection curves of the beams are illustrated in Figure 4. The load-deflection curves of hybrid FRP-steel RC beams can be approximated into three linear segments, meaning that sudden changes in slope took place at two points along the curve. The first point corresponds to the formation of the first flexural crack in the beam, i.e., the first cracking point. The second point corresponds to the initiation of the yielding of steel tension reinforcement. Figure 4 indicates that the reduction in the slope after the initiation of flexural cracking decreases with the increasing proportion of steel within the total tension reinforcement. Similarly, the load at the initiation of yielding increases, i.e., the second slope reduction takes place at higher load levels, with an increasing steel proportion in the total reinforcement. Different from the hybrid FRP-steel and steel RC beams, the FRP RC beams undergo a slope reduction only at the initiation of the flexural cracking. Beyond the initiation of cracking, the load-deflection curve follows an almost linear path up towards the failure as FRP reinforcement does not yield.

Figure 4 indicates another important feature related to the load-deflection curves of hybrid steel-FRP and only FRP RC beams. Accordingly, the load-deflection curves of FRP RC beams possess numerous minor fluctuations, implying that the load capacity undergoes sudden small reductions throughout the course of loading. The fluctuations are visible on the load-deflection curves, as the curves do not progress along a straight line but rather there are minor zigzags on the curves. These fluctuations originate from the slip of the fibers inside the matrix and they are not present in the load-deflection curves of hybrid FRP-steel RC beams, indicating that the presence of steel tension reinforcement regulates the load-deflection behavior by preventing the capacity loss during the fiber slip.

**Figure 5:**
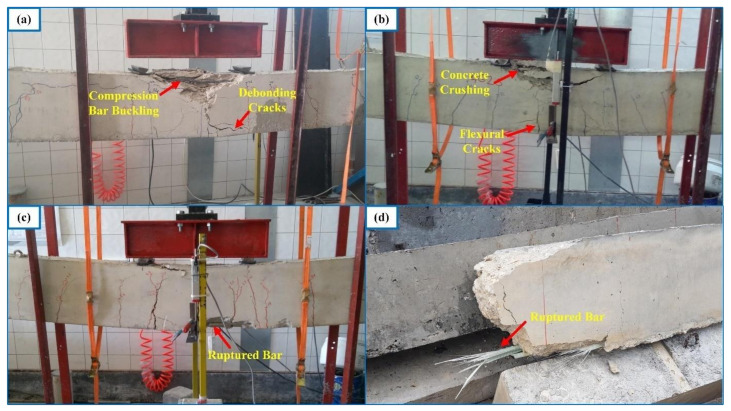
Failure modes of the specimens: compression failure (**a**) in the presence of compression bars; (**b**) in the absence of compression bars; (**c**) tension failure; and (**d**) ruptured bar.

**Figure 6:**
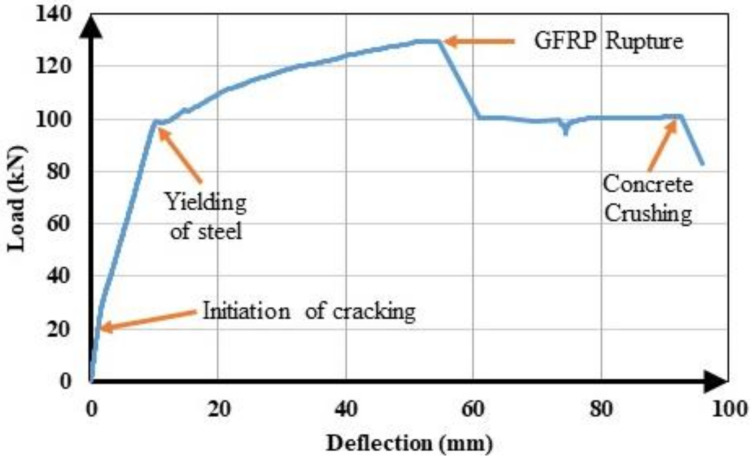
Complete load-deflection curve of G1S4.

All of the beams in the first group underwent compression failure (Figure 5a), with the exception of B1S4 and G1S4; these two beams underwent tension failure. In the complete load-deflection curve of beam G1S4 (Figure 6), the initiation of tension steel yielding, the FRP rupture, and the final failure due to concrete crushing are clearly visible. The FRP rupture can be seen to precede the concrete crushing. In B1S4 and G1S4, FRP bars ruptured almost simultaneously with the initiation of concrete crushing. Therefore, their test behavior did not differ significantly from the expected behavior

The hybrid beams in the second group experienced compression failure (Figure 5b). The ultimate load values of the beams in this group can be seen to be in close agreement (Figure 4). The test results of the first and second groups of beams clearly indicate that the beams in the same group reached close to the ultimate load values as long as they failed in the compression-controlled mode, i.e., due to the crushing of concrete before FRP rupture.

The third group of beams underwent tension failure (Figure 5c,d) as planned in the design stage. Beams B1S2 and G3 in this group, however, experienced compression failure. Despite being designed to have close ultimate loads, the beams in the third group did not reach approximate load capacities. The reference beams G3 and B5, in particular, had considerably different ultimate bending moment capacities than the other beams in the group. These differences stem from the fact that the mechanical properties of FRP bars govern the beam behavior to a major extent when a beam with hybrid FRP-steel or only FRP reinforcement is under-reinforced in terms of FRP. The entire stress-strain curve of FRP reinforcement is utilized up towards the failure and the beam fails due to the FRP rupture in this case. Hence, differences in the mechanical properties create greater differences in member behaviors and bending moment capacities in under-reinforced beams.

Variations in material properties, tensile strength, and elastic modulus values are unavoidable in FRP bars, even if they are produced from identical components and by the same supplier. Any deviations in the alignment of fibers inside the matrix or changes in the proportions of components can result in significant changes in mechanical properties, unlike steel bars, which are manufactured from a homogeneous and isotropic material. For these reasons, the beams in the third group were more liable to the variations in the ultimate load as compared to the over-reinforced, i.e., the first and second groups of beams. These unexpected variations in the mechanical properties of FRP bars can also be held responsible for the failure modes of certain test beams (B1S4, G1S4, B1S2, and G3) to deviate from the expected failure modes in the design stage.

## 5. First-Cracking Moment Calculations

The first-cracking moment is the moment at which flexural cracking initiates in an RC bending member. There are two main parameters affecting this moment. The first is the moment of inertia at the initiation of cracking and the second is the flexural tensile strength of concrete, i.e., the modulus of rupture. The present section explains the correct definitions of the moment of inertia and modulus of rupture to be used for calculating the cracking moments of RC beams with hybrid steel-FRP, only FRP, and only steel reinforcement.

### 5.1. Moment of Inertia

The moment of inertia at the initiation of cracking can be calculated by either ignoring or accounting for the contribution of longitudinal reinforcement. The gross moment of inertia (*I_g_*) is the second moment of area of the solid and homogeneous cross-section. *I_g_* ignores the contribution of the longitudinal reinforcement. The uncracked transformed moment of inertia (*I_ucr_*) includes the contribution of the longitudinal reinforcement by transforming it into an equivalent concrete area. In hybrid FRP-steel RC beams, the transformation of each of the FRP and steel reinforcement is realized according to the modular ratio of the respective reinforcement to concrete.

### 5.2. Modulus of Rupture

Three different modulus of rupture values were used for calculating the cracking moment of each beam. In addition to the measured (experimental) modulus of rupture value from the material tests on concrete prisms, two analytical values from the related equations of two international concrete codes [31,32] were adopted. Eurocode 2 [31] recommends the following equation for calculating the mean flexural tensile strength of concrete (*f_ctm,fl_*):

(11) $$f_{ctm,fl}=\max\left\{\left(1.6-h/1000\right)f_{ctm}\;;f_{ctm}\right\}$$

where *h* is the total member depth in mm and *f_ctm_* is the mean direct tensile strength calculated from

(12) $$f_{ctm}=0.30\times f_{ck}^{2/3}\leq C50/60$$

where *f_ck_* shows the characteristic compressive strength of concrete. According to this equation, the flexural tensile strength of the two concrete mixes were calculated as 3.87 and 3.81 MPa, respectively. Next, ACI 318M-19 [32] gives the following modulus of rupture (*f_r_*) expression:

(13) $$f_{r}=0.623\sqrt{{f^{\prime }}_{c}}$$

where *f*′*_c_* is the specified concrete compressive strength. The modulus of rupture was obtained as 3.48 and 3.44 MPa for the two concrete mixes.

### 5.3. Comparison of the Analytical and Experimental Cracking Moment Values

A total of six analytical cracking moment values were calculated for each beam by using two different definitions for the moment of inertia, namely *I_g_* and *I_ucr_*, and three different definitions for the modulus of rupture, namely the measured value from the material tests (experimental) and the calculated values from the two codes [31,32]. The cracking moments (*M_cr_*) are obtained by dividing the product of the respective uncracked moment of inertia and modulus of rupture with the distance from the outermost tension layer to the centroid of the gross or transformed section. The experimental (test) cracking load of a beam (*P_crt_*) refers to the first point on the load-deflection curve at which a sudden reduction in the slope is observed. The experimental and analytical cracking load values of all the test beams are presented in Table 2 and Table 3. *P_cr_*_1_ and *P_cr_*_2_ in Table 2 are the cracking load values calculated by using the experimental modulus of rupture together with *I_ucr_* and *I_g_*, respectively. Similarly, *P_cr_*_1*E*_ and *P_cr_*_2*E*_ refer to the cracking load estimates according to the Eurocode 2 [31] modulus of rupture formula in combination with *I_ucr_* and *I_g_*, respectively. Finally, *P_cr_*_1*A*_ and *P_cr_*_2*A*_ correspond to the cracking load estimates according to the ACI 318M-19 [32] modulus of rupture formula in combination with *I_ucr_* and *I_g_*, respectively.

The beams are presented in four groups in Table 2 and Table 3, namely only steel-reinforced, only FRP-reinforced, and hybrid FRP-steel over and under-reinforced beams. The experimental-to-estimated cracking load ratios of the beams are tabulated together with the mean value and percent coefficient of variation of this ratio for each beam group. Among the four different beam groups, the highest agreement between the analytical and experimental cracking load values was achieved in hybrid FRP-steel RC beams, particularly in the over-reinforced ones. The experimental values significantly exceeded the analytical estimates in beams with only steel reinforcement, even when the experimental modulus of rupture was used in the calculations. *P_cr_*_1_, *P_cr_*_1*E*_, and *P_cr_*_1*A*_ values were in much closer agreement with the *P_crt_* values in steel RC beams when compared to the *P_cr_*_2_, *P_cr_*_2*E*_, and *P_cr_*_2*A*_ estimates. To be more specific, the use of *I_ucr_* in the analytical cracking moment calculations rather than *I_g_* produced much closer cracking load estimates. Ignoring the contribution of steel tension reinforcement to the uncracked moment of inertia results in greater deviations of the analytical estimates from the experimental ones due to the high modulus of elasticity of steel. The mean value of *P_crt_*/*P_cr_*_1_ was about 20% closer to unity than the respective value of *P_crt_*/*P_cr_*_2_, implying the considerable differences between the estimated values in the presence and absence of contribution of the steel tension reinforcement. Among the analytical formulations, the use of the Eurocode 2 [31] modulus of rupture expression in combination with *I_ucr_* gave the estimates in closest agreement with the experimental cracking loads.

Contrary to the steel RC beams, the estimated values were well above the experimental values in beams with only FRP reinforcement. Even the use of the experimental modulus of rupture along with *I_ucr_* yielded estimates much larger than the respective experimental cracking load values. The differences between the *P_cr_*_1_, *P_cr_*_1*E*_, and *P_cr_*_1*A*_ values and their counterparts, i.e., *P_cr_*_2_, *P_cr_*_2*E*_, and *P_cr_*_2*A*_, were inconsiderable. Accordingly, neglecting or considering the contribution of FRP tension reinforcement to the moment of inertia at the beginning of the flexural cracking had little or no influence on the cracking moment, as the modulus of elasticity of FRP is much smaller than the respective value of steel. Among the completely analytical formulations, the closest estimates were obtained by using the ACI 318M-19 [32] modulus of rupture expression together with *I_g_*. The mean value for *P_crt_*/*P_cr_*_2*A*_ was 0.73 and the coefficient of variation was 15%.

Compared to the beams with only FRP or only steel reinforcement, the cracking moments of hybrid FRP-steel RC beams were predicted rather closely, particularly when using the experimental modulus of rupture along with *I_ucr_*. The *P_crt_*/*P_cr_*_1_ ratio had a mean value of 0.98 and a coefficient of variation of 11%, implying the small deviations of the analytical estimates from the experimental values. In general, the experimental-to-estimated cracking load ratio decreases with an increasing proportion of FRP within the tension reinforcement. For instance, the *P_crt_*/*P_cr_*_1_ ratio had a value of 1.13 for G1S5, yet it dropped gradually to 0.76 in G5S1 with an increasing portion of FRP in the tension reinforcement area. To be more comprehensible, the cracking load predictions became more conservative with the increasing amount of steel in the tension zone. The Eurocode 2 [31] modulus of rupture expression can be seen to produce closer estimates when used along with *I_g_*, while the ACI 318M-19 [32] modulus of rupture expression generates closer estimates when used together with *I_ucr_*. This conclusion is valid for both the over-reinforced and under-reinforced beam groups.

Finally, the estimated cracking load values were found to remain on the conservative side in under-reinforced hybrid beams and vice versa in the over-reinforced ones. The use of *I_ucr_* yielded lower dispersion (lower coefficient of variation) in the experimental-to-estimated cracking load ratios of the hybrid beams as compared to the use of *I_g_* in the calculations.

## 6. Deflection Calculations

The present section pertains to the formulations proposed in the present study for estimating the deflections of steel, FRP, and hybrid FRP-steel RC beams along the load-deflection curve. The accuracy of the deflection estimates in RC beams is closely related to the accuracy of estimating the flexural rigidity (*EI*). This rigidity consists of two basic parameters. The first is the modulus of elasticity (*E*), which is the resistance of the material against deformation. The second is the moment of inertia (*I*), which is the bending resistance of the section geometry. The present section is devoted to establishing the correct definitions of the moment of inertia and elastic modulus that need to be used in RC beams with different reinforcement schemes and at different stages of loading for the highest accuracy of deflection estimates.

For the aforementioned loading and support conditions of the test beams, the mid-span deflections (*δ*) can be calculated from the following equation:

(14) $$\delta =\frac{P}{48\cdot EI}\left(3L^{2}\cdot L_{ss}-4L_{ss}^{3}\right)$$

where *L* and *L_ss_* correspond to the total and shear span lengths of the beam, i.e., 2800 and 1150 mm, respectively. *I* in this equation corresponds to the uncracked value (*I_ucr_* or *I_g_*) before *M_a_* reaches *M_cr_*. Beyond *M_cr_*, *I_e_* is used in the equation. Finally, *E* was obtained from the ACI 318M-19 [32] or secant modulus definitions, summarized in Section 6.2, in the respective load-deflection curves.

### 6.1. Moment of Inertia

This section presents the moment of inertia definitions considered in the bending moment calculations of the present study. The definitions prior to and after the initiation of flexural cracking were revealed.

The main reason for the increase in deformations or decrease in the rigidity of an RC beam beyond the first-cracking point concerns the formation of new flexural cracks and propagation of the existing ones. Different definitions for the moment of inertia are used in the present section to establish the definitions that yield the closest deflection estimates throughout the course of loading.

Numerous discrete flexural cracks form in the tension zone of an RC beam as soon as the cracking moment is exceeded. The number, width, and extent of these cracks increase with the increasing load level. The cracks propagate towards the compression zone with increasing deformations in the beam. As the cracks reach the neutral axis, the section is denoted as fully cracked. The fully cracked moment of inertia (*I_cr_*) is accepted as the lowest resistance of the beam section against bending. The neutral axis depth of the beam in the fully cracked condition is denoted with *c_cr_* and calculated from the equilibrium of the first moments of the area above and below the axis.

**Figure 7:**
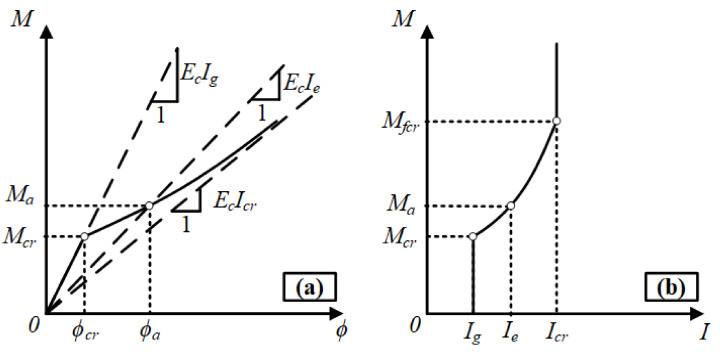
(**a**) Moment–curvature (*M*–*ϕ*) relationship of a typical RC beam with tension-controlled behavior [33]. (**b**) Variation of moment of inertia with applied moment for the same beam.

The formation of discrete cracks along the beam manifests the presence of uncracked regions between these cracks. Bond stresses develop between the rebars and concrete in these uncracked portions of the tension zone and these portions continue contributing to the moment of inertia of the beam. This contribution is referred to as tension-stiffening. With increasing bending moments in the beam, the existing cracks widen and propagate, and new cracks form in the uncracked portions. Hence, the tension-stiffening decreases and this contribution vanishes as soon as the beam reaches the fully cracked state. The gradual decrease in the moment of inertia from the uncracked value (*I_ucr_* or *I_g_*) to the fully cracked value (*I_cr_*) is reflected by the effective moment of inertia (*I_e_*). The *I_e_* expression of Branson [16] (Equation (1)) accounts for the tension-stiffening effect by providing a gradual transition between the uncracked and cracked moments of inertia (Figure 7). *M_fcr_* in this figure denotes the bending moment under which the beam section reaches the fully cracked condition, i.e., the flexural cracks reach the neutral axis.

Nevertheless, this definition is not appropriate for the FRP RC and hybrid FRP-steel RC beams, as previously explained in detail. The Bischoff [17] effective moment of inertia equation yields more realistic deflection estimates in all the steel, FRP, and hybrid FRP-steel RC beams tested in the present study, as the accuracy of the estimates from this equation is not limited to a certain uncracked-to-cracked moment of inertia range as the Branson [16] equation. For this reason, the Bischoff [17] effective moment of inertia equation (Equation (10)) was used in the present study.

**Figure 8:**
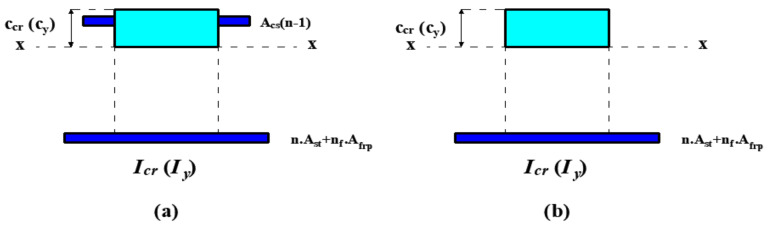
Equivalent transformed section at the fully cracked state and the initiation of tension steel yielding for beams (**a**) with and (**b**) without compression reinforcement.

If the fully cracked stage of the beam is reached before the initiation of the yielding of tension reinforcement, the effective moment of inertia shows a gradual transition from the uncracked to the fully cracked moments of inertia (Figure 8). For beams with only FRP reinforcement, the original version of the Bischoff [17] effective moment of inertia expression (Equation (10)), i.e., the transition from the uncracked to the fully cracked, was used. However, the analytical calculations indicated that the strain at the tension reinforcement of the fully cracked stage was greater than the yielding strain of steel in beams with only steel or hybrid FRP-steel reinforcement. In other words, the fully cracked stage succeeded the initiation of yielding. Consequently, the gradual transition in the moment of inertia took place between the uncracked stage and the initiation of steel yielding in these beams. Accordingly, the Bischoff [17] effective moment of inertia expression was rearranged for hybrid FRP-steel and only steel RC beams, as in Equation (15). *I_y_* in the equation refers to the yielding moment of inertia. *I_g_* in this equation can be substituted with *I_ucr_* based on the degree of contribution of the tension reinforcement to the moment of inertia. The compression depth at the yielding moment is expressed with the term *c_y_*. *I_y_* does not ignore the contribution of the steel tension reinforcement as this moment of inertia refers to the resistance directly prior to the yielding. Equation (16) presents the yielding moment of inertia of a hybrid FRP-steel RC beam with compression reinforcement.

(15) $$\frac{1}{I_{e}}={\left(\frac{M_{cr}}{M_{a}}\right)}^{2}\;\frac{1}{I_{g}}\;+\;\left[1-{\left(\frac{M_{cr}}{M_{a}}\right)}^{2}\right]\;\frac{1}{I_{y}}$$

(16) $$I_{y}=\left[\frac{bc_{y}^{3}}{12}+bc_{y}{\left(\frac{c_{y}}{2}\right)}^{2}\right]\;+\;\left[\left(nA_{st}+n_{f}A_{frp}\right){\left(d-c_{y}\right)}^{2}\right]\;+\;\left[\left(n-1\right)A_{sc}{\left(c_{y}-d^{\prime }\right)}^{2}\right]$$

where *n* and *n_f_* are the modular ratios of the steel and FRP bars, respectively, to concrete; *A_st_*, *A_frp_*, and *A_sc_* correspond to the areas of the tension steel, tension FRP, and compression steel reinforcement, respectively; and *d* and *d*′ correspond to the effective depths of the tension and compression reinforcement.

### 6.2. Modulus of Elasticity of Concrete

This section presents the modulus of elasticity definitions considered in the bending moment calculations of the present study. The definitions are valid along the entire load-deflection curve.

The entire beam section is transformed into an equivalent concrete area when calculating the bending deformations. Therefore, the modulus of elasticity of concrete (*E_c_*) is used in the calculations. In the present study, two different definitions of this modulus were utilized, as will be discussed in the following sections.

#### 6.2.1. ACI 318M-19 Modulus of Elasticity Expression

The ACI 318M-19 [32] code defines the modulus of elasticity of concrete as the secant modulus corresponding to 45% of the compressive strength. Accordingly, the modulus of elasticity can be obtained from the cylinder compressive strength according to the following equation:

(17) $$E_{c}=4700\sqrt{{f^{\prime }}_{c}}$$

where *f*′*_c_* is the specified cylinder concrete compressive strength in MPa.

#### 6.2.2. Secant Modulus of Elasticity

The secant modulus of elasticity is defined as the slope of the line connecting a point on the stress-strain curve to the origin. This modulus is a function of strain. The strain varies from zero at the neutral axis to a maximum value *ε_c_* at the extreme fibers of the compression zone. Hence, the modulus of elasticity changes along the depth of the compression zone when the beam is subjected to pure bending. The secant modulus (*E_sec_*) corresponding to the extreme fiber strain *ε_c_* (Equation (18)) represents the average modulus of elasticity of the entire compression zone.

(18) $$E_{\sec}=\frac{f_{c}\left(\varepsilon_{c}\right)}{\varepsilon_{c}}$$

where *f_c_*(*ε_c_*) is the stress at the outermost concrete fibers. With increasing applied load, the strain and stress values in the compression zone increase while the secant modulus decreases. The secant modulus reaches its lowest value at the peak point on the curve, which is the specified compressive strength.

### 6.3. Analytical Calculations

The analytical curve of each beam was obtained by calculating the deflections corresponding to a total of 20–25 preselected load values. Since the moment of inertia expression changes beyond the initiation of cracking and initiation of yielding in hybrid RC beams, the cracking and yielding points were specified on the curve. By using the appropriate effective moment of inertia and modulus of elasticity expressions, the deflections corresponding to the preselected load values were determined from Equation (14) and the curve was drawn using these points.

**Figure 9:**
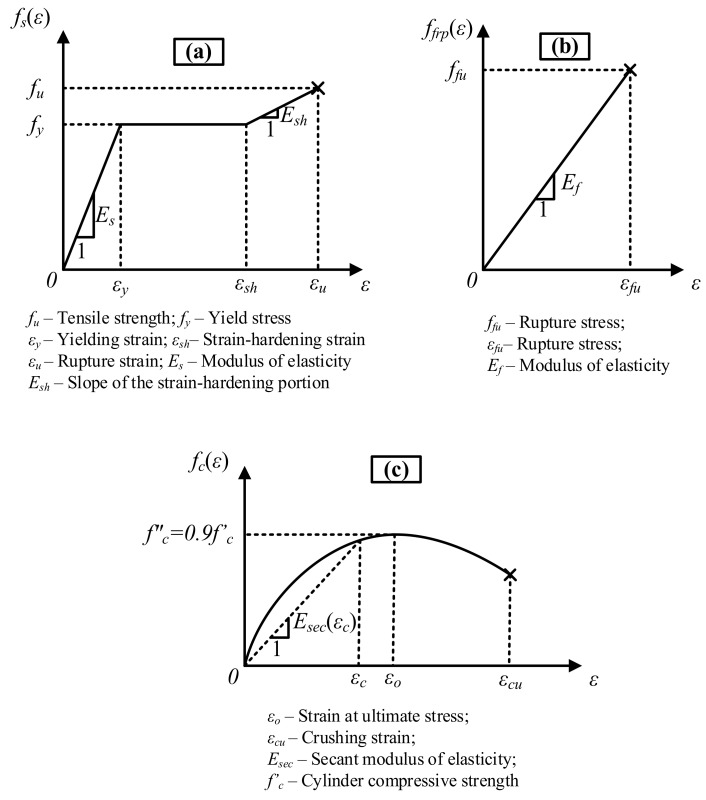
Stress-strain curves for (**a**) steel under tension; (**b**) FRP under tension; and (**c**) concrete under compression.

In the secant modulus of elasticity calculations, FRP and steel tension reinforcement were assumed to be subjected to identical strains throughout the course of loading, i.e., both types of bars were assumed to have a full bond with the surrounding concrete. The stress-strain model of Todeschini et al. [34] for concrete, trilinear, and bilinear stress-strain models, which are for tension, compression steel, and idealized linear stress-strain relationships regarding FRP tension reinforcement, were adopted in the analytical calculations (Figure 9). The minor deviations of the stress-strain curve of FRP from linearity were neglected.

In the trilinear stress-strain model of mild steel, the yielding and strain-hardening portions of the curve start at the yielding strain (*ε_y_*) and at a strain value of 0.005 (*ε_sh_*), respectively. The slopes of the elastic and strain-hardening portions of the curve were taken as 200 GPa and 1.5 GPa, respectively, based on the material tests. The ultimate strength and rupture strain of FRP are expressed as *f_fu_* and *ε_fu_*, respectively (Figure 9).

Todeschini et al. [34] express the entire stress-strain curve of concrete, i.e., both of the ascending and descending portions of the curve, with a single continuous function:


(19) $$f_{c}(\varepsilon_{c})=\frac{2\cdot f_{c}^{\prime \prime }\cdot \left(\frac{\varepsilon_{c}}{\varepsilon_{o}}\right)}{1+{\left(\frac{\varepsilon_{c}}{\varepsilon_{o}}\right)}^{2}}$$

In this model, the maximum compressive stress (*f_c_*″) is assumed to be equal to 90% of the cylinder compressive strength (*f*′*_c_*). *ε_o_* shows the strain value corresponding to the maximum stress and is calculated from the following formula:


(20) $$\varepsilon_{o}=1.71\cdot \frac{f_{c}^{\prime }}{E_{c}}$$

The crushing strain of concrete (*ε_cu_*) was taken as 0.0038. In addition, the coefficients *β*_1_(*ε_c_*) and *k*_2_(*ε_c_*) for the equivalent width of the rectangular compression block and the depth of its centroid from the compression face are obtained from the following equations:


(21) $$\beta_{1}\left(\varepsilon_{c}\right)=\frac{\ln\left[1+{\left(\frac{\varepsilon_{c}}{\varepsilon_{o}}\right)}^{2}\right]}{\frac{\varepsilon_{c}}{\varepsilon_{o}}}$$

(22) $$k_{2}\left(\varepsilon_{c}\right)=1-\frac{2\cdot \left[\left(\frac{\varepsilon_{c}}{\varepsilon_{o}}\right)-a\tan\left(\frac{\varepsilon_{c}}{\varepsilon_{o}}\right)\right]}{{\left(\frac{\varepsilon_{c}}{\varepsilon_{o}}\right)}^{2}\cdot \beta_{1}\left(\varepsilon_{c}\right)}$$

Furthermore, concrete was assumed to have a linear stress-strain curve up to the rupture under tensile stresses from bending.

**Figure 10:**
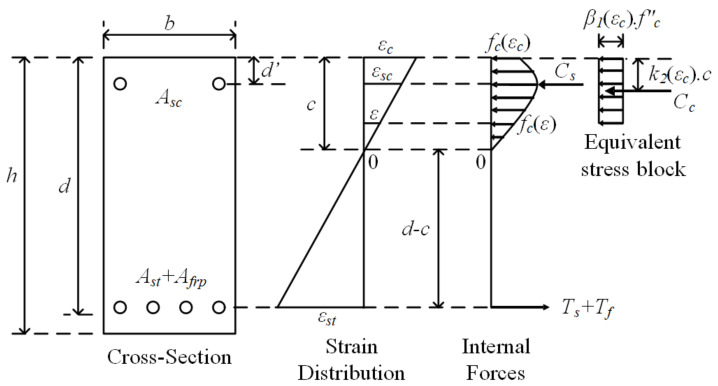
Strain and stress distributions in the section.

The secant modulus of elasticity for each preselected load (*P_s_*) value depends on the extreme compression fiber strain at this load. In the present study, an iterative approach was used to calculate the neutral axis depth (*c*) and the strain at the outermost concrete fibers (*ε_c_*) for the selected load. In this approach, first, a value was assigned to *ε_c_* and the strains of both the tension reinforcement (*ε_st_*) and compression reinforcement (*ε_sc_*) were expressed in terms of *ε_c_* according to the following equations (Figure 10):


(23) $$\varepsilon_{st}=\varepsilon_{c}\cdot \frac{d-c}{c}$$

(24) $$\varepsilon_{sc}=\varepsilon_{c}\frac{c-d^{\prime }}{c}$$

Next, using *ε_c_*, *ε_st_*, and *ε_sc_* in the following equations, the total tension and compression forces in the section were calculated:

(25) $$T=T_{s}+T_{f}=A_{st}\cdot f_{s}\left(\varepsilon_{st}\right)+A_{frp}\cdot f_{frp}\left(\varepsilon_{st}\right)$$

(26) $$C=C_{c}+C_{s}=0.9\cdot f_{c}^{\prime }\cdot b\cdot \beta_{1}\left(\varepsilon_{c}\right)\cdot c+A_{sc}\cdot f_{sc}\left(\varepsilon_{sc}\right)$$

where *T_s_*, *T_f_*, *C_s_*, and *C_c_* correspond to the total forces of the steel tension reinforcement, FRP tension reinforcement, steel compression reinforcement, and concrete rectangular compression block, respectively. *f_s_*(*ε_st_*), *f_frp_*(*ε_st_*), and *f_sc_*(*ε_sc_*) indicate the stress in the tension steel and FRP reinforcement corresponding to *ε_st_*, and the stress in the compression steel corresponding to *ε_sc_*, respectively. The stress values were calculated from the respective strains using the stress-strain models in Figure 9. Finally, *β*_1_(*ε_c_*) was calculated from Equation (21).

From the equality of internal tension and compression forces, the neutral axis depth corresponding to the extreme compression fiber strain was obtained. By using the internal forces and neutral axis depth from the above equations, the contributions of the tension reinforcement (*M_t_*), concrete compression block (*M_cc_*), and compression reinforcement (*M_sc_*) to both the moment capacity and bending moment (*M_b_*) corresponding to the extreme compression fiber strain *ε_c_* were obtained as follows:


(27) $$\begin{array}{l} M_{b}=M_{t}+M_{cc}+M_{sc}\\ =\left(T_{s}+T_{f}\right)\cdot \left(d-c\right)+C_{c}\cdot \left(c-k_{2}\left(\varepsilon_{c}\right)\cdot c\right)+C_{s}\left(c-d^{\prime }\right) \end{array}$$

*k*_2_(*ε_c_*) in this equation is calculated from Equation (22). The load corresponding to *ε_c_* was obtained from the following equation based on the loading and support conditions of the beam:


(28) $$P_{s}=\frac{2\cdot M_{b}}{L_{ss}}$$

*L_ss_* shows the shear span of the beam. The iterations were repeated until the load calculated from Equation (28) was equal to the preselected load. Upon reaching the equality of the load values, the secant modulus of elasticity was calculated from the ratio of the stress *f_c_*(*ε_c_*) corresponding to *ε_c_* to the strain *ε_c_* itself. *f_c_*(*ε_c_*) was obtained from Equation (19).

### 6.4. Comparison of the Analytical and Experimental Load-Deflection Curves

#### 6.4.1. Hybrid FRP-Steel and Steel RC Beams

In conventional steel RC beams, the deformations can be accurately estimated up to the yielding of tension reinforcement, as both steel and concrete remain roughly in the elastic regions of their respective stress-strain curves. The analytical deflections from Equation (14) are in close agreement with the experimental values within the elastic limits. The section loss in the member due to flexural cracking, however, was taken into consideration by using the Bischoff [17] effective moment of inertia expression. Beyond the yielding of steel, the deformations increase excessively and cannot be accurately estimated due to the plasticity of the reinforcing material. Hence, a horizontal analytical curve is adopted beyond the yielding point, implying an infinite increase in deflections with yielding.

In hybrid FRP-steel RC beams, steel tension bars yield before the beam reaches its bending capacity. In beams with high proportions of FRP in the total tension reinforcement, steel bars yield and their elastic material behavior terminates at rather earlier stages of loading. Nonetheless, FRP reinforcement maintains its elasticity up to the ultimate bending capacity even after the yielding of steel. The findings of Yoon et al. [35] also substantiate these assumptions.

**Figure 11:**
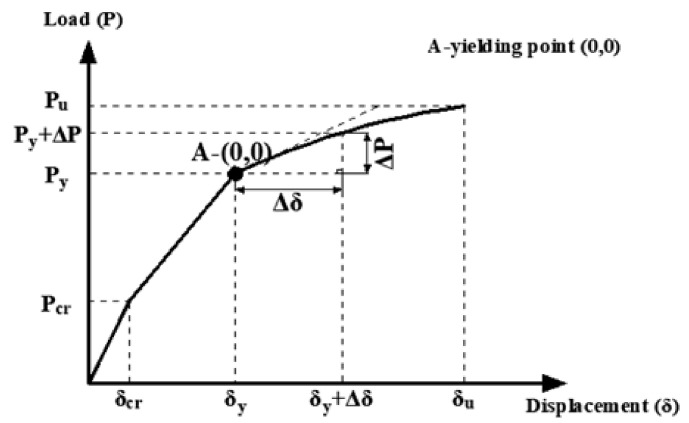
Method for estimating deflections of hybrid FRP-steel RC beams.

Based on the early yielding of steel and the preservation of the elastic material behavior of FRP up to the failure, a new method was developed and applied in the present study for estimating deflections of hybrid FRP-steel RC beams (Figure 11). According to this method, deflections of hybrid RC beams are estimated from identical equations as for steel RC beams until the tension-steel yielding. After the yielding point, the method neglects the contribution of the steel reinforcement as the elastic modulus of steel vanishes with the yielding. The deflection calculations in this phase rely on the assumption that the loading restarts after the yielding. In other words, this new method accepts the yielding point (point A in Figure 11) as the starting point for a new loading phase and estimates the deflections from a new elastic curve equation. In the new equation, incremental deformations (Δ*δ*) are calculated for incremental loads (Δ*P*) with the help of Equation (14). The total load (*P*) and deflection (δ) of the beam are obtained by adding these incremental quantities to the yielding load (*P_y_*) and yielding deflection (*δ_y_*). The moment of inertia of the beam right before the initiation of the yielding is called the yielding moment of inertia (*I_y_*). However, at the start of the reloading (point A), the response of the beam is characterized by the second yielding moment of inertia (*I_y_*_2_) rather than by *I_y_*. *I_y_*_2_ neglects the contribution of tension steel, unlike *I_y_*. Beyond the initiation of the yielding, the effective moment of inertia gradually decreases from *I_y_*_2_ to the moment of inertia of the fully cracked state. The fully cracked moment of inertia expression in hybrid RC beams should neglect the contribution of the steel reinforcement due to the fact that yielding takes place before the section reaches the completely cracked condition. Hence, a new definition was adopted in the present study for the fully cracked moment of inertia, namely the second fully cracked moment of inertia (*I_cr_*_2_). Accordingly, the Bischoff [17] equation is rearranged as follows:

(29) $$\frac{1}{I_{e}}={\left(\frac{M_{cr}}{M_{a}}\right)}^{2}\cdot \;\frac{1}{I_{y2}}\;+\;\left[1-{\left(\frac{M_{cr}}{M_{a}}\right)}^{2}\right]\;\cdot \;\frac{1}{I_{cr2}}$$

where *I_y_*_2_ and *I_cr_*_2_ can be obtained from the following equations.

(30) $$I_{y2}=\left[\frac{bc_{y}^{3}}{12}+bc_{y}{\left(\frac{c_{y}}{2}\right)}^{2}\right]\;+\;\left[\left(n_{f}A_{frp}\right){\left(d-c_{y}\right)}^{2}\right]\;+\;\left[\left(n-1\right)A_{sc}{\left(c_{y}-d^{\prime }\right)}^{2}\right]$$

(31) $$I_{cr2}=\left[\frac{bc_{cr}^{3}}{12}+bc_{cr}{\left(\frac{c_{cr}}{2}\right)}^{2}\right]\;+\;\left[\left(n_{f}A_{frp}\right){\left(d-c_{cr}\right)}^{2}\right]\;+\;\left[\left(n-1\right)A_{sc}{\left(c_{cr}-d^{\prime }\right)}^{2}\right]$$

where *c_cr_* corresponds to the neutral axis depths at the fully cracked condition. The terms with *A_cc_* (compression reinforcement) in the equations are ignored in the absence of compression reinforcement. The original definition (*I_cr_*) considers the contribution of tension steel considering that reaching the fully cracked conditions generally precedes the initiation of yielding in regular RC beams. The proposed analytical method for the estimation of deflections of hybrid RC beams is summarized in Table 4.

Different alternatives for the elastic modulus and moment of inertia in each range of loading, i.e., from the start to first cracking (0–*P_cr_*), from first cracking to yielding (*P_c_*_r_–*P_y_*), and from yielding to the ultimate load (*P_y_*–*P_ult_*), are presented in the table along with the deflection type from the elastic curve equation in each phase.

**Figure 12:**
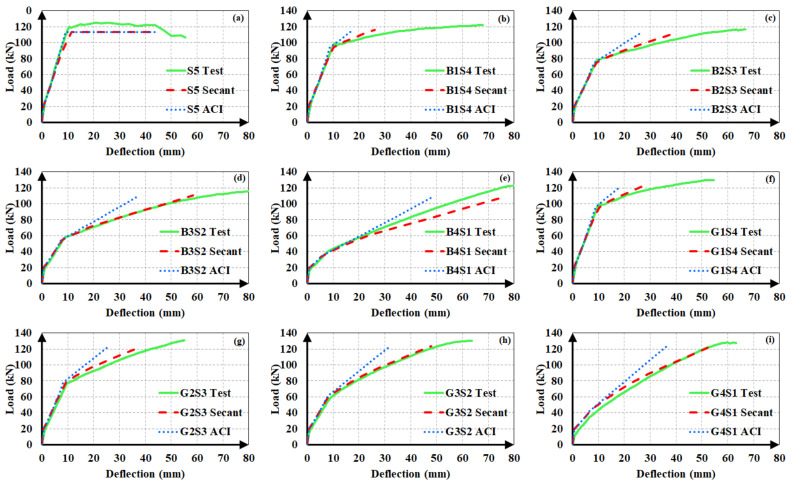
Experimental and analytical load-deflection curves of the first set of beams. (**a**) S5; (**b**) B1S4; (**c**) B2S3; (**d**) B3S2; (**e**) B4S1; (**f**) G1S4; (**g**) G2S3; (**h**) G3S2 and (**i**) G4S1.

**Figure 13:**
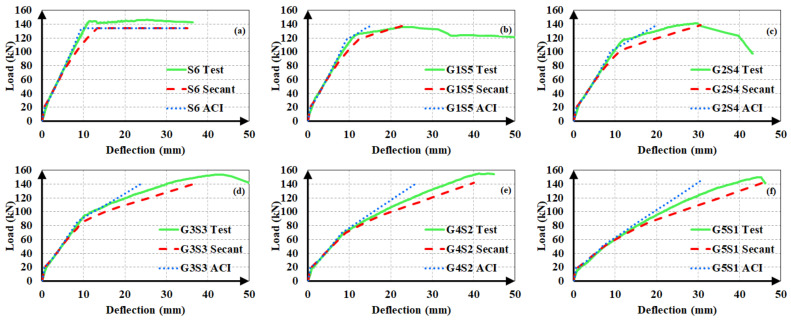
Experimental and analytical load-deflection curves of the second set of beams. (**a**) S6; (**b**) G1S5; (**c**) G2S4; (**d**) G3S3; (**e**) G4S2 and (**f**) G5S1;.

**Figure 14:**
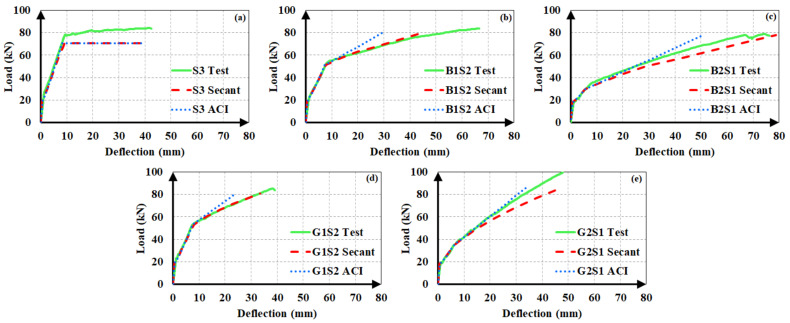
Experimental and analytical load-deflection curves of the third set of beams. (**a**) S3; (**b**) B1S2; (**c**) B2S1; (**d**) G1S2 and (**e**) G2S1.

The experimental load-deflection curves of the steel and hybrid FRP-steel RC beams are compared to the estimated ones in Figure 12, Figure 13 and Figure 14. Each of the plots contain the experimental curve and two analytical curves, which differ in the modulus of elasticity. One of the analytical curves was obtained by using the secant modulus of elasticity, while the other corresponds to the ACI 318M-19 [32] modulus of elasticity definition. In both of the analytical curves, *I_ucr_* was preferred. These plots aim at determining the modulus of elasticity definition that yields the estimates with the highest accuracy. After establishing the correct modulus of elasticity definition, the uncracked moment of inertia expression generating the closest estimates can be determined.

The plots clearly indicate that the use of the secant modulus provides closer deflection estimates to the experimental values. The two modulus of elasticity expressions have little or no influence on the calculated deflection values up to the initiation of yielding. Nevertheless, the distinction between the estimated values becomes more pronounced in the third loading phase of the curve, i.e., beyond yielding. Furthermore, the estimates, according to the secant modulus of elasticity, always remain on the safe side by exceeding the experimental values, as compared to the estimates according to the ACI 318M-19 [32] definition. This allows the material stiffness to change based on the extreme compression fiber strain along the course of loading yields to safer and closer deflection estimates in hybrid FRP-steel RC beams. For these reasons, the secant modulus should be preferred over the ACI 318M-19 [32] equation, particularly in hybrid FRP-steel RC beams. A general conclusion cannot be reached from the figures on the relationship between the proportion of FRP in the tension reinforcement and the accuracy of the deflection estimates.

In steel RC beams, however, the estimates from the two expressions barely differ and therefore both elastic modulus definitions can be used.

**Figure 15:**
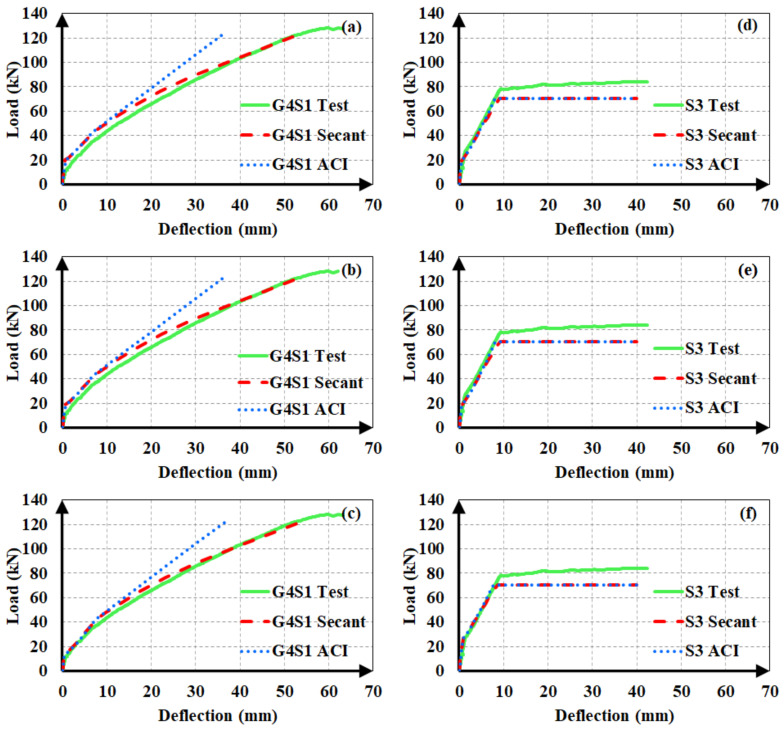
Load-deflection curves based on the analytical cracking moments from (**a**,**d**) the uncracked transformed cross-section; (**b**,**e**) gross cross-section; and (**c**,**f**) experimental cracking moments.

After establishing that the use of the secant modulus of elasticity yields more accurate and conservative estimates, the authors sought the moment of inertia expression for the uncracked stage that generates closer analytical deflections. Accordingly, the use of *I_g_* or *I_ucr_*, i.e., ignoring or accounting for the longitudinal reinforcement, was found to have minor influence on the calculated deflection values in hybrid RC beams as long as the same cracking moment was used in the calculations. If *M_cr_* is also calculated according to the uncracked moment of inertia definition, the difference between the estimates from *I_g_* and *I_ucr_* increases majorly. Figure 15 illustrates the influence of the first-cracking moment on the analytical deflection predictions for a hybrid FRP-steel (G4S1) and a reference steel RC (S3) beam. Three plots were considered for each of the beams. In one of these graphs (Figure 15c,f), the experimental load-deflection curve was compared to the analytical curve obtained by using the experimental cracking moment along with the secant or ACI 318M-19 [32] modulus of elasticity definitions. Figure 15a,d depict the analytical curves corresponding to the cracking moment calculated by using *I_ucr_*, while Figure 15b,e illustrate the curves according to the cracking moment from *I_g_*. When calculating *M_cr_* from *I_g_* or *I_ucr_*, the centroids of the gross and uncracked transformed sections, respectively, were adopted. The Bischoff [17] effective moment of inertia expression was used in all of the analytical curves. Figure 15 clearly indicates that the accuracy of the estimates can be markedly improved by the use of the experimental cracking moment in the equations. In particular, there is a rather close agreement between the experimental and analytical deflection values of hybrid RC beams when the secant modulus of elasticity and experimental cracking moment are preferred in the analytical calculations. The accuracy of the first-cracking load directly affects the fit between the experimental and analytical load-deflection curves. When this load is not correctly estimated, the portion of the load-deflection curve beyond the cracking point diverges from the experimental curve and non-conservative deflection estimates are calculated. Among the two uncracked moment of inertia expressions, *I_ucr_* yields closer cracking moments and therefore more accurate deflection estimates. In steel RC beams, however, the use of experimental cracking moments create little or no influence on the deflection estimates. The close estimation of the yielding point seems to be more significant in the agreement between the analytical and experimental curves of steel RC beams.

#### 6.4.2. FRP RC Beams

The deflections of FRP RC beams are estimated using the original expression of Bischoff [17], i.e., Equation (10). In the deflection calculations, both modulus of elasticity expressions were used. Since there was no steel reinforcement in the tension zone, *I_y_* and *I_y_*_2_ were not used in these beams. Beyond the initiation of cracking, the effective moment of inertia changes gradually from *I_ucr_* or *I_g_* to *I_cr_*. After reaching the fully cracked state, though, two different methods can be applied. These methods are denoted as Model A and Model B in the following discussion.

The first model, i.e., Model A, is based on the same procedure as the deflection calculations of hybrid RC beams in the post-yielding stage. To clarify, this method accepts the full-cracking point as the origin for the restart of a new loading phase (such as point A in Figure 11). Beyond this point, incremental deflections (Δ*δ*) are calculated for incremental loads (Δ*P*). The total load (*P*) and deflection (δ) values are obtained by adding these incremental values to the load (*P_fcr_*) and deflection (*δ_fcr_*) values at the full-cracking point. This model is summarized in Table 5. Different alternatives for the elastic modulus and moment of inertia in each range of loading, i.e., from the start to first cracking (0–P_cr_), from first cracking to full cracking (*P_cr_*–*P_fcr_*), and from full cracking to the ultimate load (*P_fcr_*–*P_ult_*), are tabulated together with the deflection type from the elastic curve equation in each phase.

The second method, i.e., Model B, differs from Model A in the deflection calculations beyond the full-cracking point. As shown in Table 6, the same procedure was applied in the *P_cr_*–*P_fcr_* and *P_fcr_*–*P_ult_* phases of loading in this method and no incremental deflection values were obtained for the last stage of loading.

**Figure 16:**
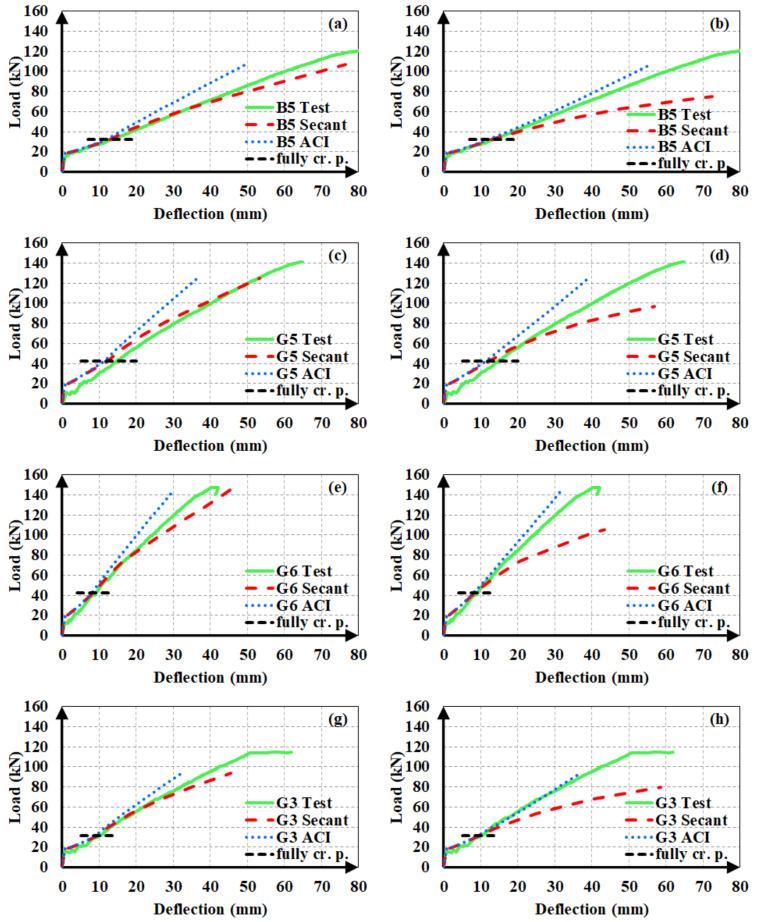
Load-deflection estimates of FRP RC beams from (**a**,**c**,**e**,**g**) Model-A and (**b**,**d**,**f**,**h**) Model-B.

Figure 16 illustrates the experimental load-deflection curves of FRP RC test beams with the analytical curves, obtained from two different modulus of elasticity expressions and two different models. In all analytical curves, *I_g_* was adopted as the moment of inertia in the uncracked stage, i.e., prior to the initiation of flexural cracking. The figure clearly shows that the agreement between the analytical and experimental deflections increases when Model A is used in calculations as well as the secant modulus of elasticity. In other words, calculating incremental deflections from load increments beyond the full-cracking point gives many superior estimates as compared to calculating the total deflections along the course of loading, particularly when using the secant modulus. The analytical estimates from the ACI 318M-19 [32] modulus of elasticity expression were constantly smaller than the experimental values, implying that this expression overvalues the material stiffness, particularly in the later stages of loading.

The ACI 318M-19 [32] definition provides closer deflection estimates when used in combination with Model B. The use of the secant modulus of elasticity in Model B resulted in excessive and over-conservative deflection values once the section was fully cracked. Accordingly, the secant modulus of elasticity should be preferred when applying Model A, yet the ACI 318M-19 [32] definition should be adopted when using Model B.

**Figure 17:**
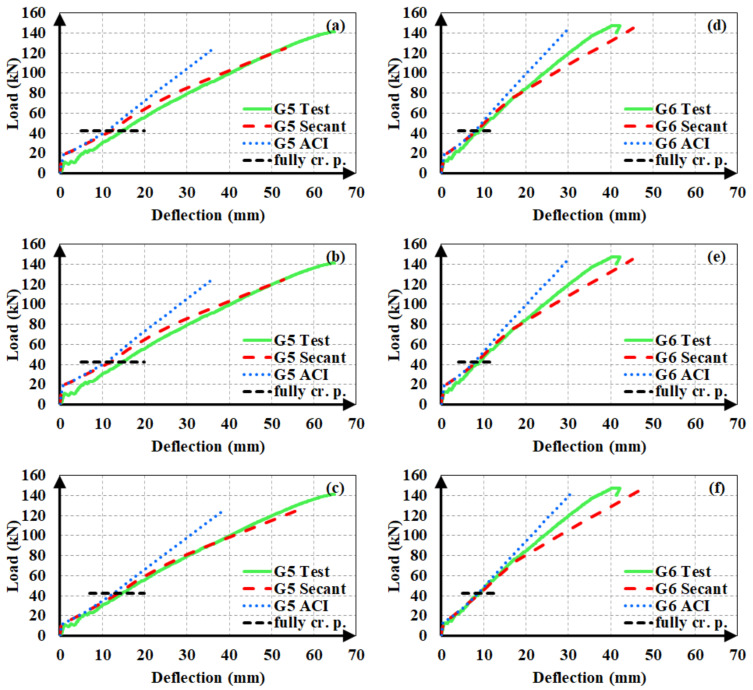
Load-deflection estimates of beams G5 and G6 using the analytical cracking moments from (**a**,**d**) gross; (**b**,**e**) uncracked transformed cross-sections; and (**c**,**f**) experimental cracking moments.

After deciding that Model A yields closer deflection estimates compared to Model B, the effect of the cracking moment on the accuracy of analytical estimates was evaluated. The experimental load-deflection curves of beams G5 and G6 were compared to the analytical curves obtained by using the experimental cracking moment values and two analytical values according to the gross and uncracked transformed cross-sections, as depicted in Figure 17. Model A was used in all analytical calculations. The use of the experimental cracking moment increased the accuracy of the analytical estimates to a major extent. The two analytical cracking moment values can be seen to have slight influence on the estimated deflections. The agreement of the analytical estimates with the experimental values increased limitedly when the cracking moment was obtained from uncracked transformed sections rather than gross sections.

## 7. Conclusions

The load-deflection behavior and cracking moments of RC beams with hybrid FRP-steel, only steel, and only FRP reinforcement were investigated. For this purpose, a total of 25 beams were tested under four-point bending. The hybrid RC beams were designed as over and under-reinforced in terms of FRP to examine the deflections of beams with different types of failures. The FRP and hybrid FRP-steel RC beams contained two types of FRP bars, namely GFRP and BFRP. The reference steel RC and FRP RC beams enabled the researchers to examine and evaluate the behavior of hybrid RC beams more comprehensively.

The mechanical properties of concrete, FRP, and steel were determined through material tests. Furthermore, prismatic samples were tested under four-point bending to determine the modulus of rupture of concrete. In cracking moment calculations, two types of uncracked moment of inertia, namely gross and uncracked transformed, were adopted. Furthermore, three alternative modulus of rupture values were employed in the calculations, namely the experimental modulus of rupture and the analytical values from the equations in Eurocode 2 [31] and ACI 318M-19 [32].

Analytical methods were proposed for the estimation of deflections of hybrid FRP-steel and FRP RC beams. Two types of moduli of elasticity, namely the secant modulus and ACI 318M-19 [32] modulus, were considered in the calculations. The gradual transition between the moments of inertia at the start and finish points of each region in the load-deflection curve was provided by the Bischoff [17] effective moment of inertia expression. The same two definitions, i.e., the gross and uncracked transformed moments of inertia, were used to reflect the sectional rigidity in the uncracked stage of the beam. The test results of the beams were analyzed and discussed in detail. The most important findings of the study can be summarized as follows:

- The load-deflection curve of a hybrid FRP-steel RC beam can be approximated into three linear segments, separated by the initiation of flexural cracking and initiation of yielding. The slope of the curve undergoes sudden reductions as soon as the flexural cracking initiates at the first-cracking moment and the tension steel starts yielding at the yielding moment. As the steel proportion within the tension reinforcement increases, tension steel yields under greater loads and the beam undergoes smaller deflections at the same load levels. The deformation capacity of a hybrid RC beam increases with increasing FRP content in the tension zone.
- The sole presence of FRP in the tension zone results in fluctuations along the load-deflection curve. These fluctuations correspond to minor losses of load capacity in the FRP reinforcing bars, caused by the slip of fibers inside the matrix. The fluctuations are not encountered in hybrid FRP-steel RC beams. In other words, the fiber slip does not affect the beam behavior when steel bars and FPR bars are used in the tension zone.
- Among different beam groups, the analytical cracking moment values were in closest agreement with the experimental values in over-reinforced hybrid RC beams. The least agreement between the experimental and analytical cracking moment values, however, corresponds to the beams with only FRP reinforcement. The variations in the mechanical properties of FRP bars, due to the variations in the material composition and the undesired but unavoidable misalignment of fibers inside the matrix, complicate the estimation of the cracking moments of FRP RC beams. These uncertainties decrease the accuracy of the estimates.
- The use of the uncracked transformed moment of inertia increases the accuracy of the analytical cracking moment estimates by about 20% in steel RC beams as compared to the use of the gross moment of inertia. Accordingly, the contribution of longitudinal steel to the response of the beam in the uncracked stage should not be ignored when the beam is reinforced solely with steel bars. In beams with only FRP reinforcement, however, ignoring or considering the contribution of longitudinal reinforcement, i.e., using the uncracked transformed or gross moment of inertia, has little influence on the accuracy of the analytical estimates. This insignificant influence is related to the low modulus of elasticity of FRP, as compared to steel.
- In both under-reinforced and over-reinforced hybrid RC beams, the cracking moments can be quite closely estimated when the Eurocode 2 [31] modulus of rupture expression is used, alongside when the gross moment of inertia or the ACI 318M-19 [32] modulus of rupture expression is used in combination with the uncracked transformed moment of inertia.
- The cracking moments of FRP RC beams are significantly overestimated by the analytical formulations, while the cracking moments of steel RC beams are markedly underestimated by these formulations even when the experimental modulus of rupture is used in calculations. The accuracy of the analytical estimates in FRP RC beams can be increased by using the ACI 318M-19 [32] modulus of rupture expression together with the gross moment of inertia.
- The secant modulus of elasticity corresponding to the extreme compression fiber strain provides closer and safer deflection estimates in hybrid FRP-steel RC beams, particularly after the initiation of yielding in the tension steel. The deflection estimates based on the ACI 318M-19 [32] modulus of elasticity expression are generally significantly lower than the experimental values. In other words, the decrease in the material stiffness in the course of loading cannot be reflected in the calculations when the ACI 318M-19 [32] modulus definition is used.
- The deflections in hybrid RC and FRP RC beams along the entire load-deflection curve should not be calculated directly from the elastic curve equation. Instead, these beams should be assumed to undergo a new loading phase (reloading) at the initiation of yielding or once the beam reaches the fully cracked stage. In other words, the yielding or full-cracking points should be assumed to be the origin for a loading phase of hybrid and FRP RC beams, respectively. In this new loading phase, deflection increments are calculated from load increments and the total deflections are obtained from the summation of both these deflection increments and the deflection at the start of the new loading phase. In this way, the load-deflection curves of the hybrid FRP-steel and FRP RC beams were shown to be estimated more closely.
- The success of the analytical deflection calculations depends on the accurate estimation of the cracking moment of a beam. The accuracy of the estimates can be greatly increased by using the experimental cracking moment of a beam. The present tests indicated that the cracking moment from the uncracked transformed section yields closer deflection estimates in comparison with the estimates from the gross cross-section.

Within the scope of the present study, the deflection behavior and cracking moments of concrete beams with hybrid GFRP-steel and BFRP-steel reinforcement were investigated. BFRP bars had a ribbed surface and GFRP bars had a surface with wire wrapping to increase the bond strengths of the bars in concrete. FRP bars are manufactured with various types of fiber (aramid, basalt, carbon, and glass), resin (polyester, epoxy, and vinyl ester), and surface finish types (smooth, braided, ribbed, wound, wrapped, and sand-coated). Further experiments on the bar (AFRP and CFRP) and surface finish types, which were not included in the present study, might be useful for validating the findings of the present study.

## Figures and Tables

**Table 1:** Details and mechanical properties of the test beams.

Group	Specimen	Sectional Dimensions, mm	Tension Reinforcement	Concrete Strength, MPa	FRP Modulus of Elasticity, GPa Mean (Standard Deviation)	FRP Tensile Strength, MPa Mean (Standard Deviation)
First	S5	200 × 300	5*Ø*12 steel	31.28	-	-
	B1S4	200 × 300	1*Ø*8.68 BFRP + 4*Ø*12 steel	31.28	43 (1.0)	1034 (33)
	B2S3	200 × 300	2*Ø*8.68 BFRP + 3*Ø*12 steel	31.28	43 (1.0)	1034 (33)
	B3S2	200 × 300	3*Ø*8.68 BFRP + 2*Ø*12 steel	31.28	43 (1.0)	1034 (33)
	B4S1	200 × 300	4*Ø*8.68 BFRP + 1*Ø*12 steel	31.28	43 (1.0)	1034 (33)
	B5	200 × 300	5*Ø*8.68 BFRP	31.28	43 (1.0)	1034 (33)
	G1S4	200 × 300	1*Ø*12.86 GFRP + 4*Ø*12 steel	31.28	35 (3.1)	449 (61)
	G2S3	200 × 300	2*Ø*12.86 GFRP + 3*Ø*12 steel	31.28	35 (3.1)	449 (61)
	G3S2	200 × 300	3*Ø*12.86 GFRP + 2*Ø*12 steel	31.28	35 (3.1)	449 (61)
	G4S1	200 × 300	4*Ø*12.86 GFRP + 1*Ø*12 steel	31.28	35 (3.1)	449 (61)
	G5	200 × 300	5*Ø*12.86 GFRP	31.28	35 (3.1)	449 (61)
Second	S6	199.8 × 303.29	6*Ø*12 steel	30.49	-	-
	G1S5	200.8 × 301.86	1*Ø*12.23 GFRP + 5*Ø*12 steel	30.49	46 (2.7)	580 (55)
	G2S4	199.8 × 301.14	2*Ø*12.23 GFRP + 4*Ø*12 steel	30.49	46 (2.7)	580 (55)
	G3S3	200.6 × 304.43	3*Ø*12.23 GFRP +3*Ø*12 steel	30.49	46 (2.7)	580 (55)
	G4S2	198.6 × 304.57	4*Ø*12.23 GFRP + 2*Ø*12 steel	30.49	46 (2.7)	580 (55)
	G5S1	200.6 × 306.00	5*Ø*12.23 GFRP + 1*Ø*12 steel	30.49	46 (2.7)	580 (55)
	G6	200.0 × 307.00	6*Ø*12.23 GFRP	30.49	46 (2.7)	580 (55)
Third	S3	200.8 × 304.71	3*Ø*12 steel	30.49	-	-
	B1S2	199.8 × 308.00	1*Ø*8.68 BFRP + 2*Ø*12 steel	30.49	43	1034 (33)
	B2S1	199.2 × 301.71	2*Ø*8.68 BFRP + 1*Ø*12 steel	30.49	43	1034 (33)
	B5	199.0 × 302.00	5*Ø*5.3 BFRP	30.49	-	-
	G1S2	198.6 × 304.86	1*Ø*12.23 GFRP + 2*Ø*12 steel	30.49	46 (2.7)	580 (55)
	G2S1	202.0 × 301.57	2*Ø*12.23 GFRP + 1*Ø*12 steel	30.49	46 (2.7)	580 (55)
	G3	198.8 × 308.71	3*Ø*12.23 GFRP	30.49	46 (2.7)	580 (55)

**Table 2:** Experimental and estimated cracking load values of RC specimens with only steel or FRP reinforcement.

Group	Beam	*P_crt_*^a^ (kN)	*P_cr_*_1_^b^ (kN)	*P_cr_*_2_^c^ (kN)	*P_crt_*/*P_cr_*_1_	*P_crt_*/*P_cr_*_2_	*P_cr_*_1*E*_^d^ (kN)	*P_cr_*_2*E*_^e^ (kN)	*P_crt_*/*P_cr_*_1*E*_	*P_crt_*/*P_cr_*_2*E*_	*P_cr_*_1*A*_^f^ (kN)	*P_cr_*_2*A*_^g^ (kN)	*P_cre_*/*P_cr_*_1*A*_	*P_cre_*/*P_cr_*_2*A*_
Only Steel	S5	25.70	22.29	18.52	1.15	1.39	24.30	20.19	1.06	1.27	21.85	18.16	1.18	1.42
	S6	27.74	21.05	17.30	1.32	1.60	24.68	20.28	1.12	1.37	22.28	18.31	1.24	1.52
	S3	23.13	19.45	17.55	1.19	1.32	22.8	20.57	1.01	1.12	20.59	18.57	1.12	1.25
			Mean (COV%)		1.22 (7.29)	1.44 (10.14)	Mean (COV %)		1.06 (5.19)	1.25 (10.05)	Mean (COV %)		1.18 (5.09)	1.40 (9.76)
Only FRP	B5	14.53	19.08	18.52	0.76	0.78	20.80	20.19	0.70	0.72	18.71	18.16	0.78	0.80
	G5	11.08	19.11	18.52	0.58	0.60	20.83	20.19	0.53	0.55	18.73	18.16	0.59	0.61
	G6	12.45	18.20	17.74	0.68	0.70	21.34	20.80	0.58	0.60	19.27	18.78	0.65	0.66
	G3	15.93	18.06	17.83	0.88	0.89	21.18	20.90	0.75	0.76	19.12	18.87	0.83	0.84
			Mean (COV %)		0.73 (17.50)	0.74 (16.55)	Mean (COV %)		0.64 (15.99)	0.66 (15.02)	Mean (COV %)		0.71 (15.65)	0.73 (15.12)

**^a^**—experimental cracking load; **^b^**—analytical estimate for the experimental modulus of rupture and *I_ucr_*; **^c^**—analytical estimate for experimental modulus of rupture and *I_g_*; **^d^**—analytical estimate for ACI318M-11 [32] modulus and *I_ucr_*; **^e^**—analytical estimate for ACI318M-11 [32] modulus and *I_g_*; **^f^**—analytical estimate for Eurocode 2 [31] modulus and *I_ucr_*; **^g^**—analytical estimate for Eurocode 2 [31] modulus and *I_g_*.

**Table 3:** Experimental and estimated cracking load values of RC specimens with hybrid FRP-steel reinforcement.

Group	Beam	*P_crt_*^a^ (kN)	*P_cr_*_1_^b^ (kN)	*P_cr_*_2_^c^ (kN)	*P_crt_*/*P_cr_*_1_	*P_crt_*/*P_cr_*_2_	*P_cr_*_1*E*_^d^ (kN)	*P_cr_*_2*E*_^e^ (kN)	*P_crt_*/*P_cr_*_1*E*_	*P_crt_*/*P_cr_*_2*E*_	*P_cr_*_1*A*_^f^ (kN)	*P_cr_*_2*A*_^g^ (kN)	*P_cre_*/*P_cr_*_1*A*_	*P_cre_*/*P_cr_*_2*A*_
Hybrid Over-reinforced	B2S3	23.00	21.02	18.52	1.09	1.24	22.91	20.19	1.00	1.14	20.60	18.16	1.12	1.27
	B3S2	20.09	20.37	18.52	0.99	1.08	22.21	20.19	0.90	1.00	19.97	18.16	1.01	1.11
	B4S1	18.02	19.73	18.52	0.91	0.97	21.51	20.19	0.84	0.89	19.34	18.16	0.93	0.99
	G2S3	20.00	21.03	18.52	0.95	1.08	22.92	20.19	0.87	0.99	20.61	18.16	0.97	1.10
	G3S2	17.62	20.39	18.52	0.86	0.95	22.23	20.19	0.79	0.87	19.99	18.16	0.88	0.97
	G4S1	18.32	19.75	18.52	0.93	0.99	21.53	20.19	0.85	0.91	19.36	18.16	0.95	1.01
	G1S5	23.09	20.41	17.22	1.13	1.34	23.93	20.19	0.97	1.14	21.60	18.23	1.07	1.27
	G2S4	22.01	19.69	17.05	1.12	1.29	23.08	19.99	0.95	1.10	20.84	18.05	1.06	1.22
	G3S3	18.85	19.62	17.50	0.96	1.08	23.00	20.51	0.82	0.92	20.77	18.52	0.91	1.02
	G4S2	19.14	18.91	17.34	1.01	1.10	22.17	20.33	0.86	0.94	20.02	18.35	0.96	1.04
	G5S1	14.17	18.70	17.68	0.76	0.80	21.92	20.72	0.65	0.68	19.79	18.71	0.72	0.76
	B1S2	20.55	19.16	17.84	1.07	1.15	22.46	20.91	0.91	0.98	20.28	18.88	1.01	1.09
			Mean (COV %)		0.98 (11.28)	1.09 (14.04)	Mean (COV %)		0.87 (10.68)	0.96 (13.39)	Mean (COV %)		0.97 (10.81)	1.07 (13.35)
Hybrid Under-reinforced	B1S4	24.39	21.66	18.52	1.13	1.32	23.61	20.19	1.03	1.21	21.23	18.16	1.15	1.34
	G1S4	22.89	21.66	18.52	1.06	1.24	23.61	20.19	0.97	1.13	21.23	18.16	1.08	1.26
	B2S1	18.84	17.76	17.07	1.06	1.10	20.82	20.01	0.90	0.94	18.80	18.06	1.00	1.04
	G1S2	19.83	18.72	17.37	1.06	1.14	21.94	20.37	0.90	0.97	19.81	18.39	1.00	1.08
	G2S1	17.32	18.07	17.29	0.96	1.00	21.18	20.27	0.82	0.85	19.12	18.30	0.91	0.95
			Mean (COV %)		1.05 (5.76)	1.16 (10.70)	Mean (COV %)		0.92 (8.61)	1.02 (14.38)	Mean (COV %)		1.03 (8.85)	1.13 (14.21)

**^a^**—experimental cracking load; **^b^**—analytical estimate for the experimental modulus of rupture and *I_ucr_*; **^c^**—analytical estimate for the experimental modulus of rupture and *I_g_*; **^d^**—analytical estimate for ACI318M-11 [32] modulus and *I_ucr_*; **^e^**—analytical estimate for ACI318M-11 [32] modulus and *I_g_*; **^f^**—analytical estimate for Eurocode 2 [31] modulus and *I_ucr_*; **^g^**—analytical estimate for Eurocode 2 [31] modulus and *I_g._*

**Table 4:** Method for estimating the deflections of hybrid FRP-steel RC beams.

Parameter	Load Range		
	0–*P_cr_*	*P_cr_*–*P_y_*	*P_y_*–*P_ult_*
Moment of inertia	*I_g_* or *I_ucr_*	Bischoff [17] expression from *I_g_* to *I_y_* or from *I_ucr_* to *I_y_*	Bischoff [17] expression from *I_y_*_2_ to *I_cr_*_2_
Modulus of elasticity	ACI 318M-19 [32] definition or secant modulus	ACI 318M-19 [32] definition or secant modulus	ACI 318M-19 [32] definition or secant modulus
Deflection from the elastic curve equation	Total deflection (*δ*)	Total deflection (*δ*)	Incremental deflection (Δ*δ*)

**Table 5:** Model A for estimating the deflections of FRP RC beams.

Parameter	Load Range		
	0–*P_cr_*	*P_cr_*–*P_fcr_*	*P_fcr_*–*P_ult_*
Moment of inertia	*I_g_* or *I_ucr_*	Bischoff [17] expression from *I_g_* to *I_cr_* or from *I_ucr_* to *I_cr_*	Bischoff [17] expression from *I_g_* to *I_cr_* or from *I_ucr_* to *I_cr_*
Modulus of elasticity	ACI 318M-19 [32] definition or secant modulus	ACI 318M-19 [32] definition or secant modulus	ACI 318M-19 [32] definition or secant modulus
Deflection from the elastic curve equation	Total deflection (*δ*)	Total deflection (*δ*)	Incremental deflection (Δ*δ*)

**Table 6:** Model B for estimating the deflections of FRP RC beams.

Parameter	Load Range	
	0–*P_cr_*	*P_cr_*–*P_ult_*
Moment of inertia	*I_g_* or *I_ucr_*	Bischoff [17] expression from *I_g_* to *I_cr_* or from *I_ucr_* to *I_cr_*
Modulus of elasticity	ACI 318M-19 [32] definition or secant modulus	ACI 318M-19 [32] definition or secant modulus
Deflection from the elastic curve equation	Total deflection (δ)	Total deflection (δ)

## Data Availability

The data will be available upon request.

## Abbreviations and Notations

FRP	fiber-reinforced polymer
AFRP	aramid fiber-reinforced polymer
BFRP	basalt fiber-reinforced polymer
CFRP	carbon fiber-reinforced polymer
GFRP	glass fiber-reinforced polymer
*A_sc_*	cross-sectional area of the compression reinforcement
*A_frp_*	cross-sectional area of the FRP tension reinforcement
*A_st_*	cross-sectional area of the steel tension reinforcement
*c_cr_*	neutral axis depth of the section at fully cracked state
*c_y_*	neutral axis depth of the section at the initiation of yielding of steel tension reinforcement
*C_c_*	internal force in the rectangular concrete compression block
*C_s_*	internal force in the compression steel
*d*	effective depth
*d*′	depth of the compression reinforcement from the compression face
*E*	modulus of elasticity
*E_c_*	modulus of elasticity of concrete
*E_f_*	modulus of elasticity of FRP
*E_s_*	modulus of elasticity of steel
*E_sec_*	secant modulus of elasticity of concrete
*E_sh_*	slope of the strain-hardening portion of the stress-strain curve of steel
*f_c_*′	specified compressive strength of concrete
*f_c_*″	maximum compressive strength of concrete in the Todeschini et al. [34] stress-strain model
*f_c_(ε_c_)*	stress in concrete corresponding to the extreme compression fiber strain
*f_ck_*	concrete characteristic compressive strength
*f_ctm_*	mean direct tensile strength of concrete
*f_ctm,fl_*	mean flexural tensile strength of concrete
*f_frp_(ε_st_)*	stress in tension FRP corresponding to the extreme compression fiber strain
*f_fu_*	rupture stress of FRP
*f_r_*	modulus of rupture of concrete
*f_s_(ε_st_)*	stress in tension steel corresponding to the extreme compression fiber strain
*f_sc_(ε_sc_)*	stress in compression steel corresponding to the extreme compression fiber strain
*h*	total beam height in mm
*I*	second moment of area (moment of inertia)
*I_cr_*	cracked moment of inertia (tension steel not yielded)
*I_cr_*_2_	second cracked moment of inertia (tension steel yielded)
*I_e_*	effective moment of inertia
*I_g_*	gross moment of inertia
*I_m_*	modified moment of inertia
*I_ucr_*	uncracked transformed moment of inertia
*I_y_*	yielding moment of inertia (right before yielding of tension steel)
*I_y_*_2_	second yielding moment of inertia (immediately after yielding of tension steel)
*k*_2_ *(ε_c_)*	coefficient in the Todeschini et al. [34] stress-strain model for the depth of the centroid of concrete compression blocks from compression faces
*L*	length of the beam span
*L_ss_*	shear span length
*m*	power term in the effective moment of inertia expression
*M_a_*	applied moment
*M_cr_*	first-cracking moment
*M_cc_*	moment contribution of concrete block
*M_fcr_*	full-cracking moment
*M_sc_*	moment contribution of compression reinforcement
*M_t_*	moment contribution of tension reinforcement
*n*	modular ratio of reinforcement to concrete
*n_f_*	modular ratio of FRP to concrete
*P*	applied load
*P_cr_*	cracking load
*P_cr_*_1_	analytical cracking load estimate for the experimental modulus of rupture and uncracked transformed moment of inertia
*P_cr_*_2_	analytical cracking load estimate for the experimental modulus of rupture and gross moment of inertia
*P_cr_*_1*A*_	analytical cracking load estimate for the ACI318m-11 [32] modulus of rupture and uncracked transformed moment of inertia
*P_cr_*_2*A*_	analytical cracking load estimate for the ACI318m-11 [32] modulus of rupture and gross transformed moment of inertia
*P_cr_*_1*E*_	analytical cracking load estimate for the Eurocode 2 [31] modulus of rupture and uncracked transformed moment of inertia
*P_cr_*_2*E*_	analytical cracking load estimate for the Eurocode 2 [31] modulus of rupture and gross transformed moment of inertia
*P_crt_*	experimental cracking load
*P_fcr_*	full-cracking load
*P_ult_*	ultimate load
*P_y_*	yielding load
*T_f_*	internal force in the tension FRP
*T_s_*	internal force in the tension steel
*α*	reduction factor in the effective moment of inertia expression
*α_b_*	reduction factor in the effective moment of inertia depending on the reinforcement ratio
*β*	reduction factor in the effective moment of inertia expression
*β_d_*	reduction factor in the effective moment of inertia reflecting the modulus of elasticity and bonding properties of FRP on beam deflections
*β*_1_ *(ε_c_)*	coefficient in the Todeschini et al. [34] stress-strain model for the width of the equivalent concrete compression block
*δ*	deflection
Δ*δ*	incremental deflection
Δ*P*	incremental load
*ε_c_*	extreme compression fiber strain
*ε_cu_*	crushing strain of concrete
*ε_o_*	concrete strain at ultimate stress
*ε_fu_*	rupture strain of FRP
*ε_sc_*	strain of the compression reinforcement
*ε_sh_*	strain of steel at the initiation of strain-hardening
*ε_st_*	strain of the tension reinforcement
*ε_y_*	yielding strain of steel
*ε_u_*	ultimate (rupture) strain of steel
*ρ*	total tension reinforcement ratio
*ρ_f_*	ratio of FRP tension reinforcement
*ρ_fb_*	balanced FRP tension reinforcement ratio
